# Indoor residual spraying with a non-pyrethroid insecticide reduces the reservoir of *Plasmodium falciparum* in a high-transmission area in northern Ghana

**DOI:** 10.1371/journal.pgph.0000285

**Published:** 2022-05-18

**Authors:** Kathryn E. Tiedje, Abraham R. Oduro, Oscar Bangre, Lucas Amenga-Etego, Samuel K. Dadzie, Maxwell A. Appawu, Kwadwo Frempong, Victor Asoala, Shazia Ruybal-Pésantez, Charles A. Narh, Samantha L. Deed, Dionne C. Argyropoulos, Anita Ghansah, Samuel A. Agyei, Sylvester Segbaya, Kwame Desewu, Ignatius Williams, Julie A. Simpson, Keziah Malm, Mercedes Pascual, Kwadwo A. Koram, Karen P. Day

**Affiliations:** 1 School of BioSciences, The University of Melbourne, at the Bio21 Molecular Science and Biotechnology Institute, Melbourne, Australia; 2 Department of Microbiology and Immunology, The University of Melbourne, at the Peter Doherty Institute for Infection and Immunity and Bio21 Molecular Science and Biotechnology Institute, Melbourne, Australia; 3 Navrongo Health Research Centre, Ghana Health Service, Navrongo, Ghana; 4 West African Centre for Cell Biology of Infectious Pathogens, University of Ghana, Legon, Ghana; 5 Parasitology Department, Noguchi Memorial Institute for Medical Research, University of Ghana, Legon, Ghana; 6 AngloGold Ashanti (Ghana) Malaria Control Programme, Obuasi, Ghana; 7 Centre for Epidemiology and Biostatistics, Melbourne School of Population and Global Health, The University of Melbourne, Melbourne, Australia; 8 Ghana National Malaria Control Programme, Public Health Division, Ghana Health Service, Accra, Ghana; 9 Department of Ecology and Evolution, University of Chicago, Chicago, IL, United States of America; 10 Epidemiology Department, Noguchi Memorial Institute for Medical Research, University of Ghana, Legon, Ghana; Tulane University School of Public Health and Tropical Medicine, UNITED STATES

## Abstract

High-malaria burden countries in sub-Saharan Africa are shifting from malaria control towards elimination. Hence, there is need to gain a contemporary understanding of how indoor residual spraying (IRS) with non-pyrethroid insecticides when combined with long-lasting insecticidal nets (LLINs) impregnated with pyrethroid insecticides, contribute to the efforts of National Malaria Control Programmes to interrupt transmission and reduce the reservoir of *Plasmodium falciparum* infections across all ages. Using an interrupted time-series study design, four age-stratified malariometric surveys, each of ~2,000 participants, were undertaken pre- and post-IRS in Bongo District, Ghana. Following the application of three-rounds of IRS, *P*. *falciparum* transmission intensity declined, as measured by a >90% reduction in the monthly entomological inoculation rate. This decline was accompanied by reductions in parasitological parameters, with participants of all ages being significantly less likely to harbor *P*. *falciparum* infections at the end of the wet season post-IRS (aOR = 0.22 [95% CI: 0.19–0.26], p-value < 0.001). In addition, multiplicity of infection (MOI_*var*_) was measured using a parasite fingerprinting tool, designed to capture within-host genome diversity. At the end of the wet season post-IRS, the prevalence of multi-genome infections declined from 75.6% to 54.1%. This study demonstrates that in areas characterized by high seasonal malaria transmission, IRS in combination with LLINs can significantly reduce the reservoir of *P*. *falciparum* infection. Nonetheless despite this success, 41.6% of the population, especially older children and adolescents, still harboured multi-genome infections. Given the persistence of this diverse reservoir across all ages, these data highlight the importance of sustaining vector control in combination with targeted chemotherapy to move high-transmission settings towards pre-elimination. This study also points to the benefits of molecular surveillance to ensure that incremental achievements are not lost and that the goals advocated for in the WHO’s High Burden to High Impact strategy are realized.

## Introduction

Recognising that malaria elimination efforts over the past decade had been largely concentrated on low transmission settings, the World Health Organization (WHO) and the Roll Back Malaria (RBM) Partnership renewed its focus on high-transmission regions in their recent High Burden to High Impact (HBHI) country-led approach in order to achieve the control and elimination targets in the Global Technical Strategy for Malaria 2016–2030 [1, 2]. In these settings the majority of the population carry infections with the malaria parasite, *Plasmodium falciparum*, without symptoms, with mainly young children and pregnant women experiencing clinical disease. Thus, the challenge for achieving elimination and ultimately eradication, is not only reducing the burden of malarial disease but reducing this large reservoir of asymptomatic infections that fuels continued transmission by *Anopheles* mosquitoes (i.e., vector) [3, 4].

In 2006, following renewed calls for elimination in sub-Saharan Africa (SSA), the WHO endorsed indoor residual spraying (IRS) with insecticide as an additional intervention for malaria control, not just for epidemic-prone areas, but specifically for regions where transmission was stable and the majority of the population was infected (i.e. holoendemic and hyperendemic) [5–7]. Long-lasting insecticidal nets (LLIN) and IRS have now become the mainstay for malaria control programmes in SSA [8]. LLINs have been shown in numerous epidemiological settings to reduce malaria morbidity and mortality, particularly among children, and have been widely distributed in SSA [9, 10]. IRS effectively reduces malaria transmission intensity by reducing mosquito population (i.e., vector) densities, but is more expensive and logistically intensive to implement, making long-term sustainability an issue [11–13].

Over the past two decades, increased funding for malaria control has led to the widespread deployment of both LLINs and IRS across much of SSA [8]. During programmatic scale up, entomological and parasitological surveillance of these interventions under operational conditions rarely occurs, and never simultaneously, as once they are found to be effective, funding to support ongoing monitoring and evaluation is time-limited. Although evidence exists supporting the role that IRS plays in reducing the burden of malaria, there are few studies from high-transmission settings that have measured the impact of IRS on the size of the *P*. *falciparum* reservoir of infection across all ages [13–20].

Ghana, located in West Africa, is an example of a country that has made impressive gains in its fight against malaria, with cases and deaths decreasing by more than half between 2005 and 2015 [1, 21]. Yet despite progress, Ghana still remains one of the 11 highest burden countries for malaria globally and is included in the WHO’s HBHI approach [2]. In 2009 the National Malaria Control Programme (NMCP) in Ghana included IRS as part of its integrated vector control strategy to reduce the burden of malarial disease, specifically in regions where *P*. *falciparum* transmission remained high [22]. Over the last decade IRS has been scaled up across much of northern Ghana against a backdrop of widely distributed LLINs through ongoing support from the United States President’s Malaria Initiative/African Indoor Residual Spraying Project (PMI/AIRS) and the AngloGold Ashanti Malaria Control Programme (AGAMal) with funding support from the Global Fund [23–25]. PMI/AIRS and AGAMal have provided considerable support for IRS procurement and implementation, as well as monitoring the impacts of IRS on entomological indicators of transmission; however, funds have not been explicitly included to concurrently monitor the impacts of IRS on malarial disease, or more specifically the reservoir of infection [26].

As high-burden countries like Ghana shift from malaria control to pre-elimination, there is an ever growing need to gain an understanding of how IRS in combination with LLINs can contribute as part of a country’s NMCP strategy in varying settings. This requires that malaria surveillance examine the efficacy of these interventions to not only decrease malaria morbidity and mortality, but also how they contribute to reducing the size and persistence of the reservoir of infection. To address this research gap, we have focused on monitoring changes in the reservoir of infection in response to an IRS intervention in Ghana as a contemporary case study for other high-transmission areas in SSA.

**Fig 1:**
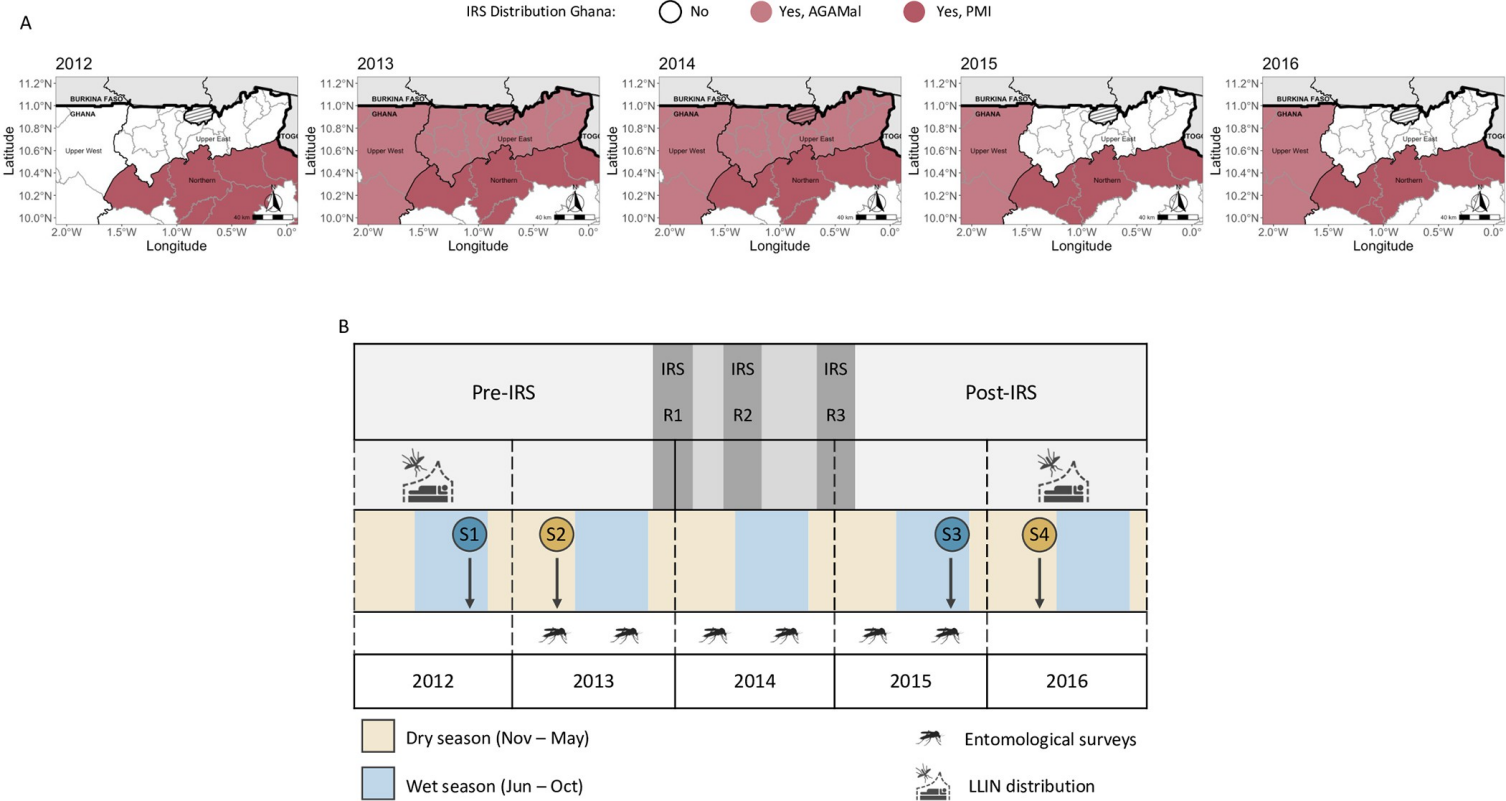
The IRS intervention and the Bongo study design. **A.** Distribution of IRS across the Upper East, Upper West, and Northern Regions of Ghana, West Africa between 2012 to 2016. Highlighted in light red are the IRS programmes funded by the Global Fund in partnership with the AGAMal (Upper East and Upper West Regions) and in dark red are those funded by PMI/AIRS (Northern Region). Districts where no shading is indicated, denote no ongoing IRS programmes during the study time period. Bongo District, where the current study took place, is denoted by the black hashed lines. The thick black lines are used to signify borders between Ghana and Burkina Faso (light grey) to the north, and Ghana and Togo (light grey) to the west. This map was drawn in R using the *rnaturalearth* package (https://github.com/ropensci/rnaturalearth) and data from Natural Earth (http://www.naturalearthdata.com/) under a CC0 license, with district-level data from the Malaria Atlas (https://github.com/malaria-atlas-project/malariaAtlas) available in an open-access repository. **B.** Four age-stratified cross-sectional surveys (N = ~2,000) were conducted in Bongo at the end of the wet (blue circles) and the end of the dry seasons (gold circles) between October 2012—June 2016. The study design can be broken down into two study phases: (1) Pre-IRS: two surveys prior to the introduction of IRS (Survey 1 October 2012 (S1); Survey 2 May/June 2013(S2)); and (2) Post-IRS: two surveys following three-rounds of IRS (Survey 3 October 2015 (S3); Survey 4 May/June 2016 (S4)). The three-rounds of IRS were implemented between 2013–2015 as indicated with grey shading: Round 1 (R1: October 2013 –January 2014, Vectoguard 40WP), Round 2 (R2: May–July 2014, Actellic 50EC), and Round 3 (R3: December 2014 –February 2015, Actellic 300C). LLINs were mass distributed in Bongo District using universal coverage campaigns between 2010–2012 and again in 2016 as indicated. The mosquitos are used to denote when the monthly entomology surveys were undertaken (February 2013 and September 2015).

As part of the AGAMal initiative in Ghana, IRS was rolled out across Bongo District in the Upper East Region between 2013 and 2015 with the aim of reducing the burden of malaria morbidity and mortality in this area characterized by high-seasonal transmission (Fig 1A). Our previous epidemiological surveillance in Bongo District, prior to the IRS intervention, identified a large asymptomatic *P*. *falciparum* reservoir across all ages and provided us with the unique opportunity to assess the impacts of adding IRS with non-pyrethroid insecticide to an area with widespread usage of LLINs (>85%) impregnated with pyrethroids [27]. By combining entomology surveys with an interrupted time-series study design we measured the impact of IRS on transmission intensity, as well as parasite prevalence, density, and multiplicity of infection (MOI_*var*_). Our study found encouraging evidence of the efficacy of this short-term IRS intervention deployed under operational conditions, to reduce the reservoir of infection in a high-transmission setting in SSA, especially when measures of within-host parasite diversity (i.e., MOI_*var*_) were considered. Taking advantage of the entomological and parasitological data collected prior to the rollout of IRS in Bongo District, we provide contemporary evidence supporting the benefits of adding IRS to LLINs to reduce both malaria transmission and the size of the *P*. *falciparum* reservoir in the human population.

## Materials and methods

This observational study was undertaken to investigate the impacts of adding IRS with non-pyrethroid insecticide to LLINs for vector control on the asymptomatic *P*. *falciparum* reservoir in Bongo District, Ghana by measuring entomological, parasitological, and parasite genetic parameters.

### Study site

Bongo District is located in the Upper East Region of Ghana and is characterized by high seasonal transmission of *P*. *falciparum* (minority *Plasmodium malariae* and *Plasmodium ovale*). Bongo District has a prolonged dry season (November—May) and a short but intense rainy season (June–October) that peaks annually in August. Detailed information on the study site and population have been previously published [27].

### Vector control interventions

LLINs impregnated with pyrethroid insecticides (i.e., PermaNet 2.0, Olyset, or DawaPlus 2.0) were mass distributed across Ghana using universal coverage campaigns (UCC), including Bongo District, by the NMCP/Ghana Health Service (GHS) between 2010–2012, approximately a year and a half prior to the IRS intervention, and again in 2016 following the discontinuation of IRS (Fig 1B) [25, 27, 28]. Between 2012 and 2016, continuous distribution of LLINs in Bongo District was undertaken through routine services like antenatal care (ANC) clinics and expanded programme on immunization (EPI) visits, as well as school distributions to maintain coverage between campaigns [25, 29]. This routine distribution of LLINs in Bongo District between 2012 and 2016 was undertaken so that net coverage remained high by the replacement and removal of damaged and/or older nets where the insecticide may no longer be bioactive (<3 years) as recommended by the WHO [29, 30]. Following the UCC in 2016, LLIN coverage in the Upper East Region was 93.9% as determined by the Malaria Indicator Survey (MIS) [25].

Starting in 2013, IRS was implemented across all of the Upper East Region, by AGAMal, using three-rounds of different organophosphate pirimiphos-methyl formulations (i.e., non-pyrethroid) (Fig 1). The first round of IRS (R1) with Vectoguard 40WP was implemented between October 2013 –January 2014 (end of the wet to the beginning of the dry season); the second round (R2) with Actellic 50EC was undertaken between May–July 2014 (end of dry to the beginning of the wet season); and finally, the third round (R3) with Actellic 300CS was between December 2014 –February 2015 (beginning/middle of the dry season). The seasonal timing of the IRS varied, particularly for the first round, which was completed at the end of the wet season when transmission was declining, rather than during or towards the end of the dry season. The irregular timing of the first round was due to the logistical challenges of insecticide availability and the implementation of this large-scale intervention across the whole district. The next two rounds of IRS were timed correctly and undertaken during the dry seasons so that the insecticides were in place prior to the emergence of the vector population. To address the technical issues around IRS timing, the third round of IRS was undertaken with Actellic 300CS, a third generation IRS (3GIRS) product that has been shown to be long-acting (~7–8 months) through bioassay experiments in northern Ghana [25, 31]. Reported IRS coverage in Bongo District, based on AGAMal operational reports was 91.8% in Round 1, 95.6% in Round 2, and finally 96.6% in Round 3 (AGAMal, personnel communication). This coverage is based on the number of eligible structures sprayed compared to the number of eligible structures encountered. In this study we also collected participant reported IRS coverage using structured questionnaires (S1 Text) and found that the average coverage for all three-rounds was 90.8% compounds (i.e., households) and ranged from 79.7% during Round 1, and increased to 96.6% and 96.1% in Round 2 and Round 3, respectively.

### Study design and procedures

Using an interrupted time-series study design, four age-stratified cross-sectional surveys of ~2,000 participants per survey were carried out between October 2012 and June 2016 to measure changes in the size of the reservoir of infection. These surveys were completed at the end of the wet season (EWS) (i.e., high-transmission season) and the end of the dry season (EDS) (i.e., low-transmission season) pre- and post-IRS (Fig 1B and S1 Table) with each survey lasting approximately 4 weeks. The study can be separated into two phases: (1) Pre-IRS: two baseline surveys conducted prior to the introduction of IRS at the EWS (Survey 1 (S1) October 2012) and at the EDS (Survey 2 (S2) May/June 2013) and (2) Post-IRS: two surveys conducted following the discontinuation of IRS at the EWS (Survey 3 (S3) October 2015) and at the EDS (Survey 4 (S4) May/June 2016) (Fig 1B). To monitor malaria control interventions throughout the study, information was collected from all participants enrolled on IRS coverage, LLIN usage the previous night, and antimalarial treatment in the previous two-weeks (i.e., participants that reported they were sick, sought treatment, and were provided with an antimalarial treatment) using structured questionnaires (S1 Table and S1 Text).

Briefly, for this study a total of 500 compounds (i.e., households) were randomly selected from each catchment area (Vea/Gowrie and Soe) in Bongo District (hereinafter referred to collectively as “Bongo”) based on the average enumeration data of 5.6 persons per compound collected in June 2012 [27]. These catchment areas were considered to be different agroecological zones (i.e., Vea/Gowrie (irrigated) vs. Soe (non-irrigated)) based on their proximity to the Vea Dam, but were otherwise similar in population size, age structure, and ethnic composition [27]. After randomly selecting an index compound in each catchment area, an equal number of male and female participants were enrolled into each of the age-stratified categories (i.e., 1–5, 6–10, 11–20, 21–39, ≥ 40 years) until the required enrolment number was reached. At the time the study was designed in 2012, *Plasmodium* spp. prevalence data in Bongo District were not available. Based on prevalence data between irrigated and non-irrigated areas from neighbouring Kassena-Nankana Municipal District, an estimated risk ratio of 3.0 during the dry season for *Plasmodium* spp. prevalence between the catchment areas was used. Therefore, at a 95% confidence level, 80% power, and sample ratio of 1:1 between the irrigated (i.e., Vea/Gowrie) and non-irrigated (i.e., Soe) catchment areas the estimated sample size per area was 865, allowing for a 15% nonresponse rate. Based on these numbers, ~1,000 participants per catchment area (i.e., ~2,000 participants total) were recruited in all surveys. This sample size of ~2,000 participants per survey was sufficient to detect a risk ratio ≤ 0.85 for *P*. *falciparum* prevalence between the pre-IRS (i.e., unexposed) and post-IRS (i.e., exposed) surveys with a 95% confidence level, 80% power, and a 1:1 sample ratio between the pre- and post-IRS surveys (EWS and EDS). Additional details on the inclusion/exclusion criteria, data collection procedures, etc. have been previously published [27].

### Entomological parameters

Monthly entomological surveys were undertaken to directly evaluate transmission: pre-IRS, during, and post-IRS. During the monthly entomological surveys, a total of four nights were used for the collections, with each catchment area being stratified into four sectors with two compounds per sector being randomly selected (i.e., 32 compounds per catchment area per month). Mosquitoes were collected using human landing catches (HLC) (indoors and outdoors) and indoor pyrethrum spray catch. All mosquitoes collected were morphologically identified to the *Anopheles* species level (i.e., *An*. *gambiae* s.l. and *An*. *funestus* group) using taxonomic keys [32]. The head and thorax for the collected female *Anopheles* spp. mosquitos were tested for the presence of *P*. *falciparum* circumsporozoite protein (CSP) using an enzyme-linked immunosorbent assay technique [33]. These *Anopheles* spp. data were then used to calculate the daily human biting-rate (HBR-daily; bites/person/night), the sporozoite rate (number of CSP positive mosquitoes/number of mosquitoes tested), and the entomological inoculation rates (EIR-daily = HBR-daily x sporozoite rate (infective bites/person/night (ib/p/n)); and EIR-monthly = EIR-daily x number of days per month (infective bites/person/month (ib/p/m))), as a measure of transmission intensity.

### Malariometric surveys

During each survey all participants enrolled completed a structured questionnaire to collect information on their demographics, socioeconomic characteristics, malaria prevention activities, recent clinical symptoms, recent antimalarial treatment, etc. In addition, body weight, blood pressure, and axillary temperature were measured. Five drops of blood were collected for thick/thin blood films, haemoglobin determination/anaemia status (as defined by the WHO) [34], and as a dried blood spot (DBS) onto filter paper (3MM Whatman, Madison, UK) for laboratory-based molecular analyses. Finally, a blood sample was screened using First Response or CareStart (*P*. *falciparum* antigen, histidine-rich protein 2 (HRP-2)) rapid diagnostic tests (RDT). Participants who had a positive RDT and were febrile (temperature ≥ 37.5°C) were considered to be symptomatic for *P*. *falciparum* and were provided with an antimalarial by clinical personnel through the Ministry of Health (MOH)/Ghana Health Service (GHS), following the current antimalarial treatment policy [35]. In addition, participants that were RDT negative and febrile but identified as *P*. *falciparum* positive by microscopy were considered to have a symptomatic infection. Approximately 2% of the participants surveyed during each of the EWS surveys (S1 = 34 and S2 = 25) and <1% during the EDS surveys (S2 = 1 and S4 = 1) met the criteria for symptomatic *P*. *falciparum* infections and were excluded from the analyses. All parasitological measurements, including *Plasmodium* ssp. identification (asexual and sexual) and density were undertaken by experienced microscopists from the Navrongo Health Research Centre as previously published [27].

#### DNA extraction

Genomic DNA was extracted from the DBS using the QIAamp DNA Mini Kit (QIAGEN, USA) according to the manufactures procedures with modifications [27]. This was undertaken for all participants (S1 to S4) with a confirmed microscopic *P*. *falciparum* infection as well as those participants in S1 (EWS, pre-IRS) and S3 (EWS, post-IRS) that were negative for *P*. *falciparum* by microscopy.

#### Molecular estimation of submicroscopic *P*. *falciparum* prevalence

For all participants in S1 (EWS, pre-IRS) and S3 (EWS, post-IRS) that were found to be negative for *P*. *falciparum* by microscopy a nested PCR targeting the 18S ribosomal RNA gene (*18S rRNA*) was performed using previously published protocols to identify those participants with submicroscopic *P*. *falciparum* infections [27].

For this study all participants who were positive for *P*. *falciparum* (including mixed *P*. *falciparum* infections) by either microscopy (i.e. microscopic *P*. *falciparum* infection) or *18S rRNA* PCR (i.e. submicroscopic *P*. *falciparum* infection), were afebrile (temperature < 37.5°C) on the day the survey was conducted, and did not report a history of fever in the 24-hours prior to being surveyed were defined as having an “asymptomatic *P*. *falciparum* infection” (hereafter designated as *P*. *falciparum* infections).

### Parasite genetic parameters

#### *Var* genotyping

For *var* genotyping or “*var*coding”, the sequences encoding the highly conserved DBLα domains of the antigen-encoding *P*. *falciparum var* genes were amplified, sequenced on a MiSeq Illumina platform, cleaned, and analysed as previously described [36–40]. This high-throughput sequencing approach was undertaken for all participants with microscopic *P*. *falciparum* infections (i.e., 2,138 isolates, S2 Table). Following the application of quality control measures, DBLα sequence data was successfully obtained from 1,776 isolates (83.1%) with 362 isolates having low quality sequencing data (i.e., < 20 DBLα types) (S2 and S3 Tables). These success rates were expected since we were working with low density asymptomatic *P*. *falciparum* infections, particularly for adults (> 20 years) and for those surveys conducted post-IRS (i.e., S3 and S4).

#### Molecular estimation of the *P*. *falciparum* multiplicity of infection

Using the *var*coding approach, we next evaluated an isolate’s multiplicity of infection (MOI) based on our understanding of the population structure of the *var* multigene family. By sequencing the DBLα domain we have previously shown that in high-transmission settings in Africa the *var* multigene family has a unique population structure that is characterized by high DBLα type diversity and limited overlap between DBLα isolate repertoires [36–38, 40]. Here we have exploited this population structure to estimate an isolate’s MOI_*var*_ (i.e., the number of genetically distinct *P*. *falciparum* genomes infecting an individual based on *var*coding) [40]. To calculate MOI_*var*_ the non-upsA DBLα types were chosen since they were ~20X more diverse and were less conserved between the isolate repertoires (i.e., isolate repertoires shared ≤ 2% of their non-upsA DBLα types) compared to the upsA DBLα types. The non-upsA DBLα repertoire size was then used to calculate the MOI_*var*_ using a cut-off value of 45 non-upsA DBLα types per *P*. *falciparum* genome. Using this cut-off isolates with repertoires ≤ 45 non-upsA DBLα types were estimated to be single genome infections (MOI_*var*_ = 1, i.e., single-genome infections), while isolates with 46 to ≤ 90 non-upsA DBLα types were estimated to be carrying two distinct *P*. *falciparum* genomes (MOI_*var*_ = 2, i.e., multi-genome infections), and so on. Using the non-upsA DBLα repertoire size, MOI_*var*_ can be defined as a continuous (i.e., non-upsA DBLα repertoire size/45) or discrete variable. In order to include those isolates with low quality sequencing data (N = 362, S3 Table) the non-upsA DBLα repertoire size was estimated to be at least 45 (i.e., MOI_*var*_ = 1), although we understand that this approximation may be an underestimate of an isolate’s true MOI_*var*_.

#### Molecular estimation of the number of *P*. *falciparum* genomes

To further estimate the number of *P*. *falciparum* genomes detected, MOI_*var*_ was summed for all isolates *var*coded in each of the four surveys (S1 to S4). For example, in S1 if we had three isolates (i.e., participant infections) with estimated MOI_*var*_ values of 2, 5, and 8 genomes, respectively, the total number of genomes for S1 would be 15.

### Statistical analysis

For these analyses participants were categorized into defined age groups, sex, catchment areas, and IRS study phases. Continuous variables are presented as medians with inter quartile ranges (IQRs) and categorical variables are presented using prevalence with 95% confidence intervals (CIs). Using the time-series data collected between 2012–2015 allowed us to measure the effect of IRS on the prevalence of *P*. *falciparum* infections (both microscopic and submicroscopic) with results expressed in terms of Attributable Risk (AR) and Attributable Risk percentage (AR%) with two-sided 95% CIs. Associations between the IRS intervention (i.e., pre- and post-IRS) and the presence of a *P*. *falciparum* infection (microscopic and/or submicroscopic) at the EWS and the EDS surveys were estimated using multivariable logistic regression. Confounding variables identified a priori (age group, sex, catchment area, LLIN usage the previous night, and antimalarial treatment in the previous two-weeks) were adjusted for in the multivariable regression models. Effect modification, identified a priori (age groups and catchment area), was investigated using an interaction term between these variables and the IRS intervention (i.e., pre- and post-IRS). Since the majority of the study participants were re-assessed at each of the four surveys the cluster sandwich variance estimator was used to deal with the correlation of these repeated measurements. All statistical analyses were performed in R (version 4.0.5) using base R and the *tidyverse* and *epiR* packages [41–43].

### Ethical approval

The study was reviewed/approved by the ethics committees at the Navrongo Health Research Centre, Ghana (NHRC IRB-131), Noguchi Memorial Institute for Medical Research, Ghana (NMIMR-IRB CPN 089/11-12), University of Melbourne, Australia (HREC 144–1986), and the University of Chicago, United States (IRB 14–1495). Individual informed consent was obtained in the local language from each enrolled participant by signature/thumbprint, accompanied by the signature of an independent witness. For children < 18 years of age a parent or guardian provided consent. In addition, all children between the ages of 12 and 17 years provided assent.

### Inclusivity in global research

Additional information regarding the ethical, cultural, and scientific considerations specific to inclusivity in global research is included in S1 Checklist.

## Results

### *P*. *falciparum* transmission post-IRS

**Fig 2:**
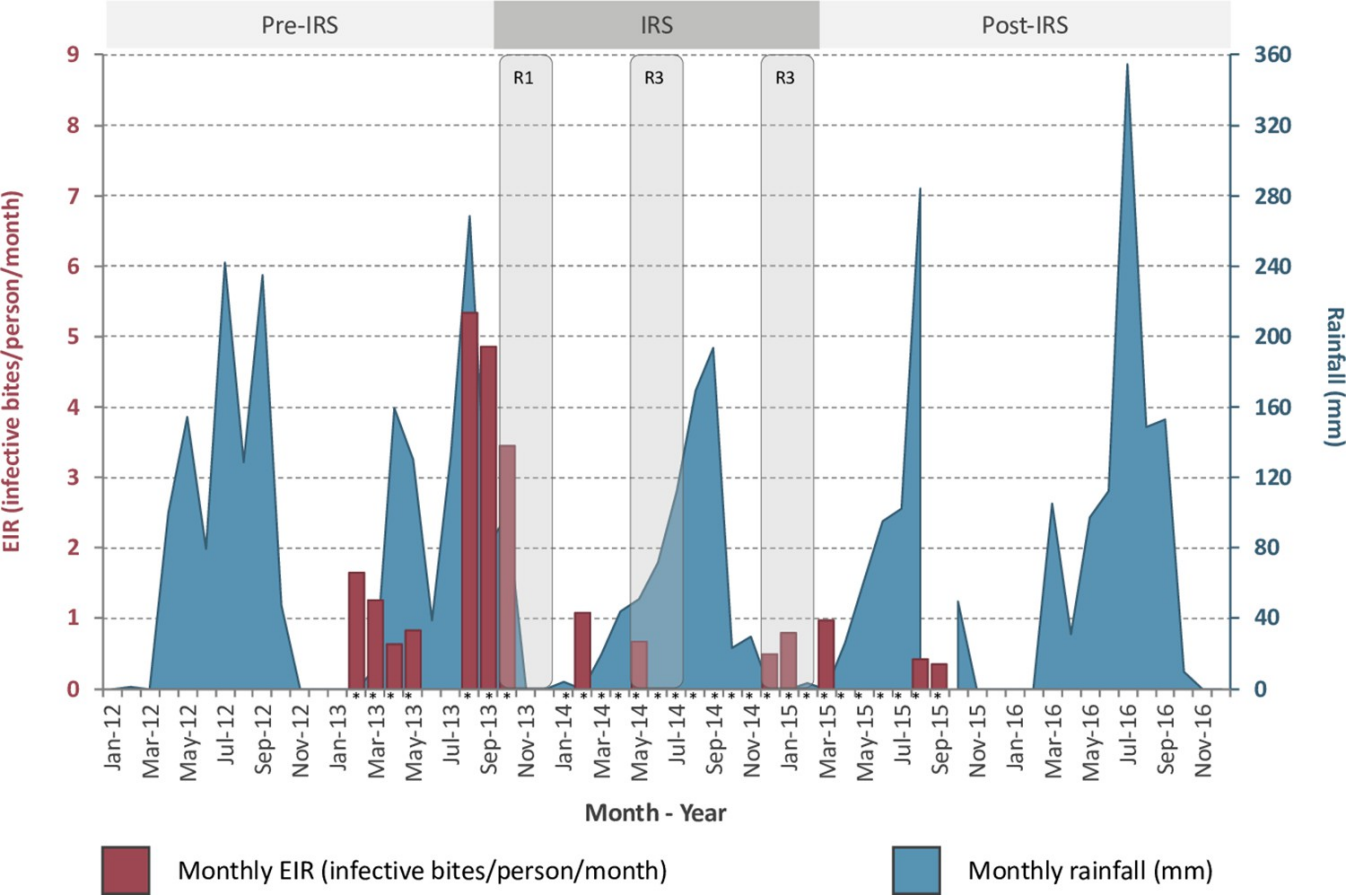
Entomological and rainfall data between 2012 and 2016. Monthly variation in the entomological inoculation rate (EIR-monthly = infective bites/person/month) (red) of *Anopheles* spp. in Bongo, Ghana in relation the three-rounds of IRS (Round 1 (R1), Round 2 (R2) and Round 3 (R3)). The asterisks (*) denote the months when the entomology surveys were completed. Due to logistical challenges, there were gaps in the monthly monitoring, however a total of 28 out of 31 months (90.3%) were covered. Since no meteorological data was available for Bongo District, the monthly rainfall data (mm) (blue) between January 2012 to December 2016 was acquired from the closest meteorological station based in Bolgatanga (~15 km south of Bongo Town by “birds flight”). The grey shading indicates the timing of the three-rounds of IRS (R1, R2, R3) between 2013–2015.

Prior to the IRS, *P*. *falciparum* transmission during the wet season, as measured by EIR (Fig 2), was substantial, despite the widespread deployment and reported usage of LLINs across all ages (S1 Table, see Materials and methods). In Bongo, IRS was deployed against this background of widespread LLIN usage. Over the course of the study self-reported LLIN usage the previous night remained high (≥ 85%) between the pre- and post-IRS surveys at the end of the wet season (EWS) and the end of the dry season (EDS) with fewer participants reporting sleeping under an LLIN at the EDS pre- and post-IRS.

In order to fully evaluate the impact of IRS with non-pyrethroid insecticide on the vector population, key entomological parameters were measured pre-, during, and post-IRS (Fig 2). Pre-IRS (February 2013 –September 2013) a total of 3,878 female *Anopheles* spp. mosquitos were collected after 384 person-nights of HLC in the two catchment areas, while during and post-IRS (October 2013 –September 2015) 2,132 were collected using 640 man-nights. The predominant vector in Bongo throughout the study period was *An*. *gambiae* s.l, with limited transmission from *An*. *funestus*. The HBR-daily varied between the dry season (April) and the wet season (August), during both the pre-IRS (4.5 b/p/n vs. 25.5 b/p/n, April 2013 and August 2013, respectively) and post-IRS (0.3 b/p/n vs. 1.8 b/p/n, April 2015 and August 2015, respectively) study phases. Following three-rounds of IRS, the HBR-daily at the peak of the wet season dropped by ~93% between August 2013 (pre-IRS) (25.5 b/p/n) and August 2015 (post-IRS) (1.8 b/p/n). Transmission of *P*. *falciparum* as expected was highly seasonal with the monthly EIRs being ~2-3x lower during pre-IRS dry season (February-May 2013, EIR-monthly range: 0.6 ib/p/m to 1.7 ib/p/m) compared to the pre-IRS wet season (August-October 2013, EIR-monthly range: 3.5 to 5.3 ib/p/m) (Fig 2). These patterns in Bongo were comparable to other studies conducted in northern Ghana and justify AGAMal’s approach to implement a single round of IRS per year during the dry season [26, 44, 45]. Following the IRS, transmission at the peak of the wet season declined by >90% between August 2013 (pre-IRS) (EIR-month = 5.3 ib/p/m) and August 2015 (post-IRS) (EIR-monthly = 0.4 ib/p/m) in Bongo. These data from a high-transmission setting in Africa, show that adding short-term IRS with non-pyrethroid insecticide to LLINs, can have significant impacts on local *P*. *falciparum* transmission intensity.

### Microscopic *P*. *falciparum* prevalence post-IRS

When we considered antimalarial treatment in the previous two-weeks, participant reported usage pre- and post-IRS was 5 times and 1.9 times higher at the EWS compared to the EDS, respectively (S1 Table). Following the IRS intervention, treatment for malaria declined at the EWS from 41.4% pre-IRS (S1) to 14.7% post-IRS (S3). While at the EDS reported antimalarial treatment remained similar between the pre- (8.1%; S2) and post-IRS (7.6%; S4) surveys. During this study antimalarial drug treatment was highest among the youngest children (1–5 years) in comparison to the other age groups surveys, both at the EWS pre-IRS (59.6% vs. ≤ 45.3%, respectively) and post-IRS (30.8% vs. ≤ 17.9%, respectively). In addition we found that following the IRS intervention fewer participants at the EWS (46.7% vs. 41.1%, S1 vs. S3, respectively) and at the EDS (32.2% vs. 28.8%, S2 vs. S3, respectively) were classified as being anaemic based on the established WHO criteria (S1 Table) [34].

**Fig 3:**
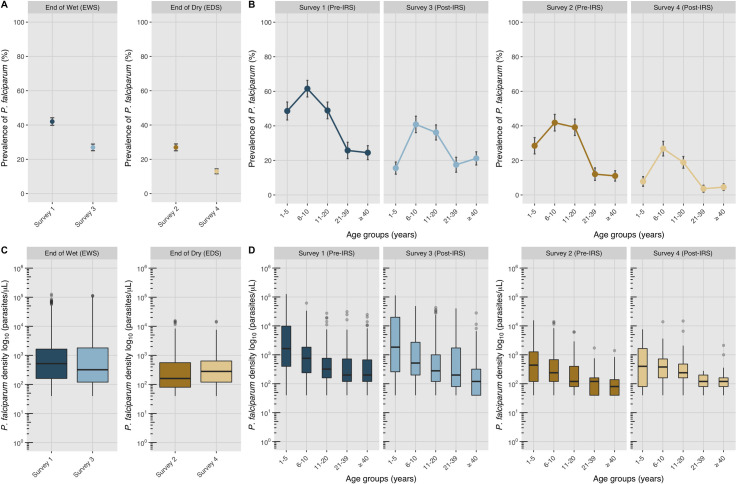
Reductions in the prevalence and density of microscopic *P*. *falciparum* infections pre- to post-IRS at the end of the wet (blue) and dry (gold) seasons. Prevalence of microscopic *P*. *falciparum* infections pre- to post-IRS at the **A.** end of the wet and dry season surveys and **B.** across each age group (years) at the end of the wet and dry season surveys pre- to post-IRS. Error bars represent the upper and lower limits of the 95% confidence interval (CI). Box and whisker plots of the log_10_-transformed microscopic *P*. *falciparum* infection densities (parasites/μL) pre- to post-IRS at the **C.** end of the wet and dry season surveys and **D.** across each age group (years) at the end of the wet and dry season surveys. The boxes represent the inter-quartile ranges (IQR) and the horizontal lines represent the median log_10_-transformed *P*. *falciparum* infection densities (parasites/μL). The whiskers are used to depict the largest and smallest log_10_-transformed infection densities and the grey dots outside the whiskers are used to denote outliers. The parasite densities were log_10_-transformed to remove skewness. For additional details see S4 Table.

To measure the impact of adding IRS to LLINs on the size of the reservoir of *P*. *falciparum* infection, four age-stratified surveys of ~2,000 participants per survey were completed pre- to post-IRS. Following the IRS, the prevalence of microscopic *P*. *falciparum* infections in Bongo declined at the EWS (42.0% to 27.0%, S1 to S3, respectively) and at the EDS (27.0% to 13.0%, S2 to S4, respectively) (Fig 3A and S4 Table). Based on these data 36.9% and 51.8% of infections that would have occurred post-IRS at the EWS (S3) and EDS (S4), respectively, were averted by this short-term IRS intervention (p*-*value < 0.001) (S5 Table). In the adjusted model, we observed that participants were 53% (aOR = 0.47 [95% CI: 0.40–0.54], p-value < 0.001) and 64% (aOR = 0.36 [95% CI: 0.31–0.43], p-value < 0.001) less likely to have a microscopic *P*. *falciparum* infection at the EWS (S3) and at the EDS (S4), respectively, compared to the pre-IRS surveys (Tables 1, S6 and S7).

**Table 1:** Association between the IRS and microscopic *P*. *falciparum* infection prevalence at the end of the wet and dry seasons.

Survey timing in relation to the IRS and seasonality		Microscopic *P*. *falciparum* infection ^a^			
		Unadjusted		Adjusted^b^	
		OR (95% CI)	p-value	aOR (95% CI)	p-value
**End of wet season (EWS)**					
	Pre-IRS (Survey 1, October 2012)	1.00	-	1.00	-
	Post-IRS (Survey 3, October 2015)	0.51 (0.45–0.58)	< 0.001	0.47 (0.40–0.54)	< 0.001
**End of dry season (EDS)**					
	Pre-IRS (Survey 2, May/June 2013)	1.00	-	1.00	-
	Post-IRS (Survey 4, May/June 2016)	0.41 (0.35–0.47)	< 0.001	0.36 (0.31–0.43)	< 0.001

For additional information on the adjusted model see S6 and S7 Tables.

OR = odds ratio; aOR = adjusted odds ratio; CI = confidence interval, to deal with the repeated measures the cluster sandwich variance estimator was used

^a^ Participants were excluded from the model if their antimalarial treatment in the previous two weeks was not known: Survey 3 (N = 79) and Survey 4 (N = 12).

^b^ Age group, sex, catchment area, LLIN usage the previous night, and anti-malarial treatment in the previous two weeks are adjusted for in the multivariable logistic regression model.

Although the addition of IRS to LLINs led to reductions in *P*. *falciparum* prevalence across all age groups (Fig 3B and S4 Table), not surprisingly the greatest impacts were observed among the youngest children (1–5 years) with 68.0% and 72.6% of the microscopic infections at the EWS (S3) and the EDS (S4), respectively, being prevented (p-value < 0.001) (S5 Table). By using this age-stratified design we were able to show that at the EWS post-IRS (S3), 40.8% and 36.2% of the older children (6–10 years) and adolescents (11–20 years), respectively, still harboured *P*. *falciparum* parasites (Fig 3B and S4 Table). However, in the older adults (≥ 40 years) at the EWS post-IRS (S3) the prevalence of microscopic infections remained comparable to that observed pre-IRS (S1) (24.5% vs. 21.2%, S1 to S3, respectively) (Fig 3B and S4 Table). Further analysis of effect modification at the EWS by age showed that IRS was associated with a significant decrease in microscopic *P*. *falciparum* prevalence for the youngest children (aOR = 0.19 [95% CI: 0.13–0.27], p-value < 0.001) and other age groups (i.e., 6–10, 11–20, and 21–39 years) (p-value ≤ 0.023), but not for the older adults (aOR = 0.83 [95% CI: 0.59–1.18], p-value = 0.311) (S8 Table). Similar magnitudes of effect were also observed by age at the EDS, they were however significant in all age groups (p-value < 0.001) (S9 Table). Thus, even after three-rounds of IRS, the prevalence of infection at the EWS and the EDS remained strongly associated with age, with the odds of microscopic parasitemia being significantly higher among older children and adolescents compared to the youngest children (p-value < 0.001, S6 and S7 Tables). This result points to the role that these particular age groups play in maintaining the *P*. *falciparum* reservoir, especially during the long dry season (Fig 3B).

### *P*. *falciparum* infection density post-IRS

To investigate this microscopically detectable *P*. *falciparum* reservoir in more detail, we next evaluated the impact of IRS on parasite density. Regardless of season (i.e., EWS or EDS) or the IRS study phase (i.e., pre- or post-IRS) the majority of infections were observed to be low (40–999 parasites/μL) to moderate (1,000–9,999 parasites/μL) densities, with <10% of infections being categorized as high-density (≥ 10,000 parasites/μL) (S1A Fig). Importantly across at the EWS (i.e., high-transmission), ~80% of these high-density infections were identified in the children (1–5 and 6–10 years) (S1B Fig).

Prior to the IRS, we found that the median *P*. *falciparum* densities ~3 times higher at the EWS (520 parasites/μL) compared to the EDS (160 parasites/μL) (Fig 3C and S4 Table). Following three-rounds of IRS, median densities declined at the EWS (520 parasites/μL vs. 320 parasites/μL, S1 vs. S3, respectively), with the distribution post-IRS (S3) being more positively skewed (i.e., lower density infections post-IRS) (Fig 3C and S4 Table). Importantly, we also found that participants at the EWS classified as anaemic carried higher-density infections compared to participants that were non-anaemic, both pre-IRS (640 parasites/μL vs. 400 parasites/μL, respectively) and post-IRS (520 parasites/μL vs. 240 parasites/μL, respectively) (S2 Fig). When we examined parasite density by age, we observed that median densities gradually declined for older participants, with the younger children (1–5 years) typically harbouring higher median density infections that were more variable (i.e., broader distributions) compared to adults (> 20 years) (Fig 3D). Although the IRS interrupted transmission, these age-specific density patterns remained consistent despite seasonality (i.e., EWS or EDS) and the IRS intervention (i.e., pre- or post-IRS).

### Submicroscopic *P*. *falciparum* prevalence post-IRS

**Fig 4:**
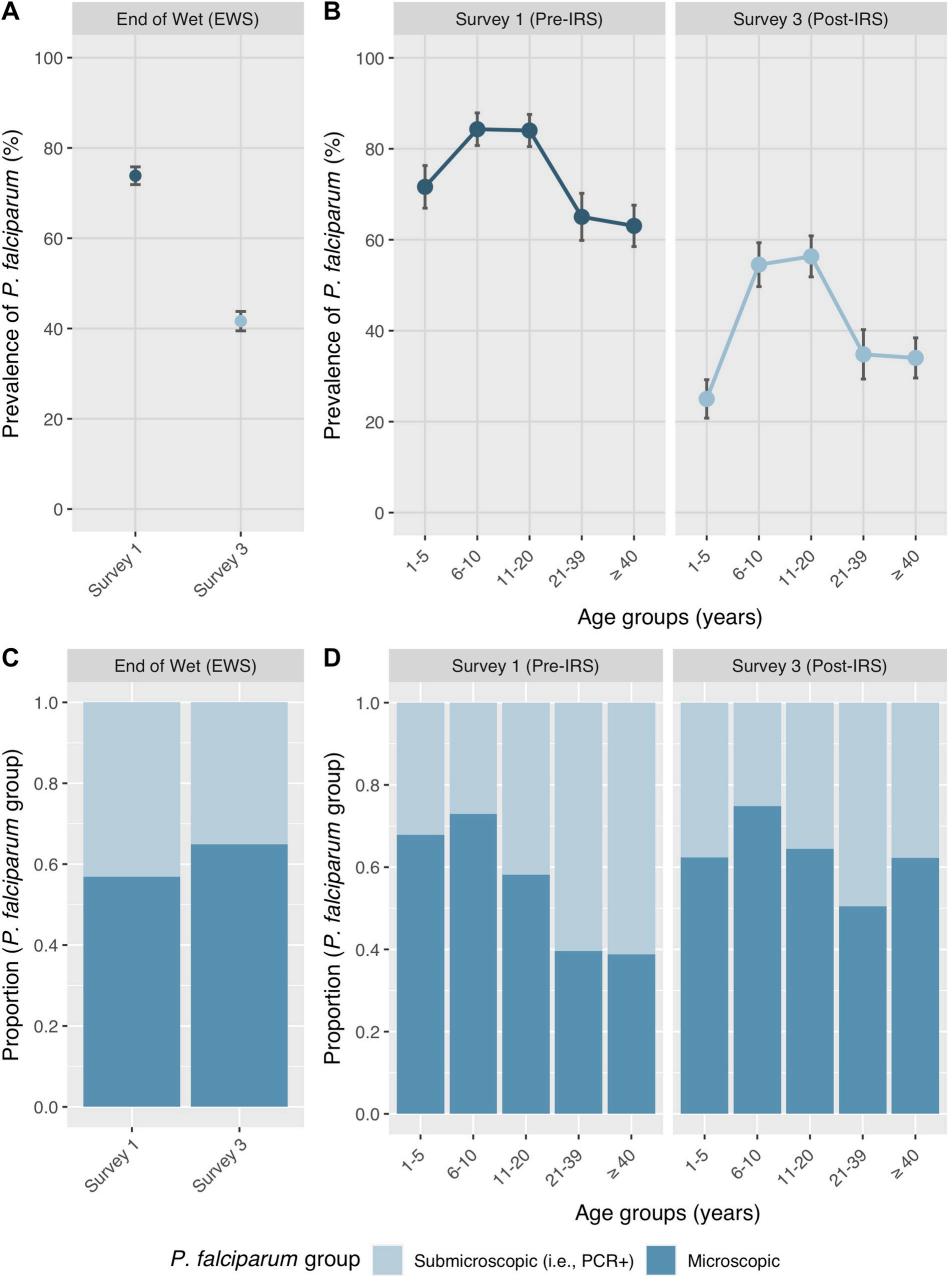
Reductions in the prevalence of microscopic and submicroscopic *P*. *falciparum* infections at the end of the wet seasons (i.e., Survey 1 and Survey 3) pre- and post-IRS. Prevalence of *P*. *falciparum* infections **A.** at the end of the wet seasons and **B.** across each age group (years) at the wet seasons pre- and post-IRS. Error bars represent the upper and lower limits of the 95% confidence interval (CI). Proportion of microscopic and submicroscopic *P*. *falciparum* infections at the **C.** at the end of the wet seasons and **D.** across each age group (years) at the end of the wet season pre- and post-IRS. Light blue bars indicate the proportion of individuals with submicroscopic *P*. *falciparum* infections detected using the *18S rRNA* PCR; while dark blue bars indicate the proportion of individuals with microscopically detectable *P*. *falciparum* infections. For additional details see S4 Table.

To better quantify the size of the *P*. *falciparum* reservoir at the EWS, especially for the older age groups where the impacts of IRS appeared negligible, sensitive molecular diagnostic methods were incorporated to evaluate the prevalence of submicroscopic *P*. *falciparum* infections (~0.05–10 parasites/μL). By including these very low-density infections, we found that the *P*. *falciparum* reservoir across all ages was substantially larger than that predicted by microscopy alone, with 73.8% and 41.6% of the of the population pre-IRS (S1) and post-IRS (S5), respectively, harbouring an infection (i.e., microscopic or submicroscopic) (Fig 4A and 4B and S4 Table). Importantly, prior to the rollout of IRS, 60.8% of the infections detected in adults (>20 years) were submicroscopic, while just under half of the infections in the adolescent population (41.8%) were submicroscopic (Fig 4C and 4D). Following three-rounds of IRS, participants were 78% (aOR = 0.22 [95% CI: 0.19–0.26], p-value < 0.001) less likely to have any *P*. *falciparum* infection (i.e., microscopic or submicroscopic) (S10 Table), with 43.6% of infections being prevented post-IRS (S3) (p-value < 0.001) (S11 Table). Again, there was evidence of effect modification by age between the IRS intervention and *P*. *falciparum* prevalence. By including the very low-density submicroscopic infections in this analysis, we observed that reductions in prevalence were significant in all age groups (p-value < 0.001), including older adults (aOR = 0.30 [95% CI: 0.22–0.41], p-value < 0.001) (S12 Table). In contrast to the microscopy results, we found that by incorporating this more sensitive diagnostic (i.e., PCR), particularly for older adults (≥ 40 years), three-rounds of IRS significantly reduced *P*. *falciparum* prevalence, with almost half of the infections in this age group being averted at the EWS post-IRS (S3) (Fig 4B and S11 Table).

### *P*. *falciparum* infection complexity post-IRS

**Fig 5:**
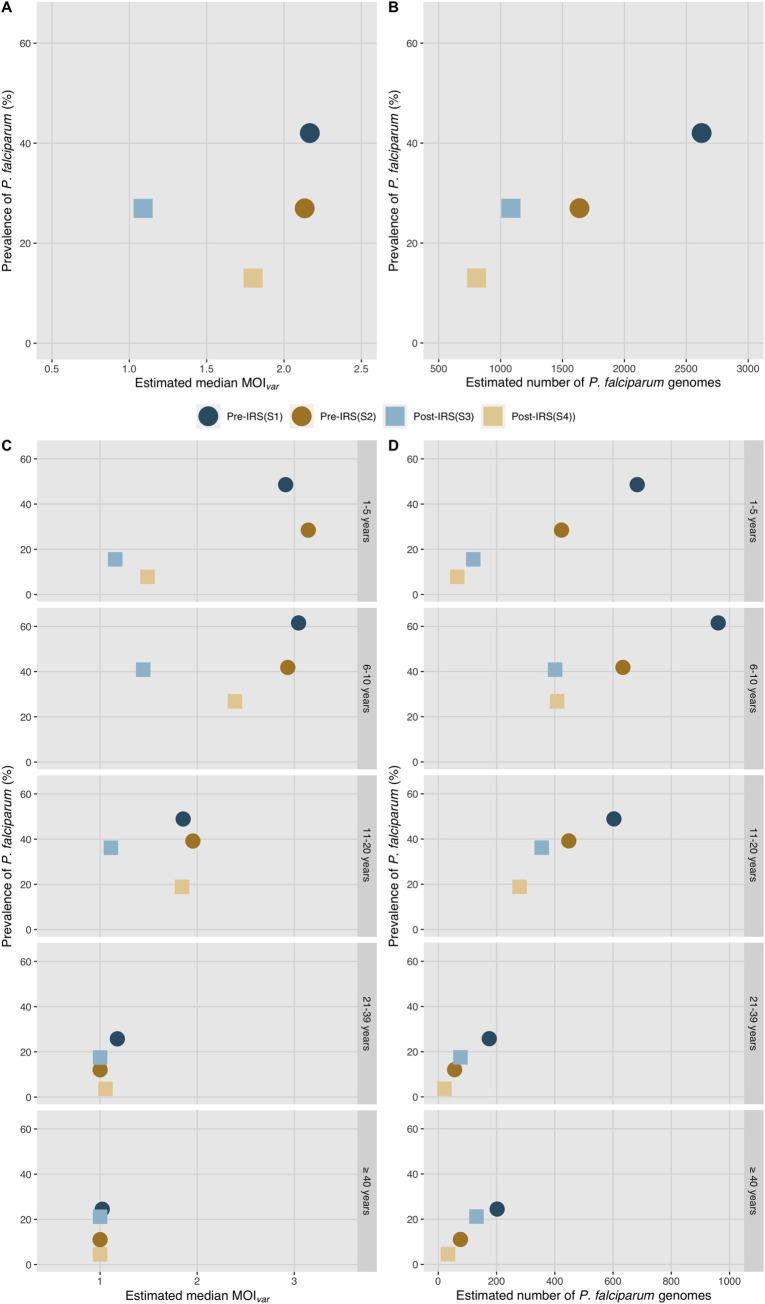
Changes in *P*. *falciparum* prevalence vs. the estimated median MOI_*var*_ and the number of *P*. *falciparum* genomes pre- to post-IRS at the end of the wet (blue) and dry (gold) seasons. *P*. *falciparum* prevalence vs. **A.** the estimated median MOI_*var*_ and **B.** the estimated number of P. falciparum genomes pre-IRS (S1 and S2; circles) and post-IRS (S3 and S4; squares*)*. *P*. *falciparum* prevalence vs. **C.** the estimated MOI_*var*_ and **D.** the estimated number of *P*. *falciparum* genomes for each age group pre-IRS (S1 and S2; circles) and post-IRS (S3 and S4; squares). For additional details see S13 Table.

Next, we evaluated the multiplicity or complexity of infection (i.e., MOI_*var*_) as an additional surrogate to monitor for changes in transmission intensity as well as the genetic composition of the *P*. *falciparum* reservoir of infection. Prior to the rollout of IRS, the majority (~75%) of participants across all ages with microscopic parasitemia carried multi-genome infections (i.e., MOI_*var*_ > 1) with the median MOI_*var*_ being comparable at the EWS (S1) and the EDS (S2) surveys (2.2 [IQR: 1.0–3.9] vs. 2.1 [IQR: 1.0–3.8], S1 vs. S2, respectively) (Figs 5A, S3A and S4A). Similar to the reductions in *P*. *falciparum* prevalence and density, median MOI_*var*_ also declined at the EWS post-IRS (1.1 [IQR: 1.0–1.8]) with fewer participants harbouring multi-genome infections (MOI_*var*_ > 1) (75.6% vs. 54.1%, S1 vs. S3, respectively). These changes in infection complexity, however, were not seen at the EDS, as median MOI_*var*_ and the proportion of multi-genome infections (i.e., MOI_*var*_ > 1) remained more stable pre- to post-IRS (74.5% vs. 75.4%, S2 vs. S4, respectively) (Figs 5A and S3A). When we examined these seasonal differences further, we found that they were largely being driven by age. Among the youngest children (1–5 years) median MOI_*var*_ declined by half between the pre- to post-IRS surveys at the EWS and the EDS (Fig 5C). Interestingly, we observed that although the median MOI_*var*_ values remained lower and more stable for the younger (1–5 years) and older (> 20 years) participants post-IRS, the opposite was found for the older children (6–10 years) and adolescents (11–20 years), as median MOI_*var*_ values started to rebound post-IRS (Fig 5C). These results show that at the end of the long dry season, the asymptomatic infections in older children and adolescents, were still composed of diverse multi-genome infections despite the success of the IRS intervention (Figs 5C, S3B and S4B).

Finally, by using MOI_*var*_ to estimate the number diverse *P*. *falciparum* genomes in the reservoir, we observed a 69.3% (i.e., 1,818 genomes lost) reduction in the number of diverse genomes in the population between the EWS pre-IRS (S1) and the EDS post-IRS (S4) (Fig 5B and S13 Table). This estimated loss in diverse genomes over the course of the IRS intervention was seen across all age groups, with the greatest overall reductions of 90.5% and 85.7% being observed in the younger children (1–5 years) and adults (>20 years), respectively (Fig 5D and S13 Table). Similar trends were also observed among older children (6–10 years) and adolescents (11–20 years) and even though the *P*. *falciparum* reservoir was smaller post-IRS, as measured by prevalence, it still persisted in these age groups and continued to be composed of multi-genome infections.

## Discussion

Here we report the results of a contemporary study of a programmatic IRS intervention against the backdrop of widespread LLIN usage. This control measure was uniquely evaluated by combining entomological, parasitological, and molecular surveillance to determine the impact of IRS on the *P*. *falciparum* reservoir in a high-transmission setting in SSA. Entomological data confirmed seasonality patterns and the success of IRS when implemented annually during the dry seasons to significantly reduce transmission. IRS also had a significant impact on the size of the reservoir of infection (i.e., microscopic and submicroscopic) in the community; with participants across all ages, sexes, and catchment areas being significantly less likely to harbor *P*. *falciparum* infections post-IRS. This observational study provides valuable evidence from a high-transmission setting that adding IRS with non-pyrethroid insecticide to LLINs, not only reduces the burden of clinical malaria [20], but also provides the added benefit of reducing the reservoir of *P*. *falciparum* infections across all ages.

For individuals residing in malaria-endemic regions, non-sterilizing immunity that protects against clinical disease is acquired through repeated exposure to *P*. *falciparum* from a young age and leads to regulation of parasite density and carriage of infections through to adulthood [46, 47]. Using microscopy, we found that the IRS had the greatest impact on the youngest participants surveyed, with 1–5 year olds being approximately three times less likely to have an infection at the end of the wet season following the IRS (i.e., Survey 3). However, in this study we also incorporated sensitive molecular diagnostic methods to specifically detect submicroscopic infections that have not been traditionally included in population-based surveys in high-transmission settings [3, 48]. Using this approach, we found that IRS had community-wide impacts on the prevalence of infection across all ages, particularly among adults (i.e., > 20 years) who carried the greatest proportion of these submicroscopic infections. These molecular data show that by not accounting for these low-density infections, which are typically found in older ages, we are underestimating the true impact of malaria control interventions like IRS, that target the whole community.

Leveraging within species diversity of *P*. *falciparum* enabled us to monitor changes in the number of diverse parasite genomes per person (i.e., MOI) in response to reduced transmission by IRS. This was achieved by selecting the most polymorphic surface antigen encoding genes (i.e., *var* genes) to determine MOI. Measuring MOI by *var*coding proved to be a sensitive and valuable metric for population-based surveys, as not only could it detect the high number of *P*. *falciparum* genomes per person (MOI_*var*_ range = 2–18), but it was also able to capture changes in MOI following the IRS intervention. This contrasts with previous studies that have focused on single copy polymorphic antigen encoding genes (e.g., *msp1*, *msp2*) and neutral loci (e.g., microsatellites and single nucleotide polymorphisms (SNPs)) as the most common approaches to estimate MOI [49, 50]. While proven to be useful in low-transmission settings targeting elimination, they have less sensitivity in high-transmission settings where the number of genomes per person exceeds the resolution of polymorphic antigen genes, microsatellites, and biallelic SNP markers.

This study validated the importance of incorporating *var*coding as a genetic approach to further monitor for changes in malaria transmission beyond *P*. *falciparum* prevalence both at the individual and the population level. Using this molecular surveillance, we observed that following three-rounds of IRS not only were participants significantly less likely to harbour multi-genome infections (MOI_*var*_ > 1), but that there was 69.3% reduction in the number of *P*. *falciparum* genomes circulating in the human population in Bongo. The reduction in MOI likely contributed to the observed reduction in *P*. *falciparum* density at the end of the wet season post-IRS. Although the impact of the IRS was observed across all age groups, the reservoir that persisted in the older children and adolescents was still largely composed of diverse multi-genome infections and perhaps reflects their ability to still tolerate multiple *P*. *falciparum* infections (i.e., superinfection) due to immunity developed prior to the IRS. To understand the impact of this short-term IRS intervention, this population should be followed up to determine whether interrupting transmission, even just temporarily, leads to changes in the development of naturally acquired immunity and shifts in the age-specific patterns of malaria infection.

## Conclusions

By combining both parasitological and molecular surveillance data, this study demonstrates that adding IRS to LLINs implemented under programmatic conditions in an area with high seasonal transmission can reduce the size of the *P*. *falciparum* reservoir across all ages. Here we also present molecular surveillance methodologies that are sensitive to age, seasonality, microepidemiology, and can document incremental changes in the reservoir of infection in high transmission. These methods can support the evaluation of sustained vector control and/or chemotherapy programmes in order to optimise the use of limited financial resources available for NMCPs in SSA to shift from malaria control to pre-elimination and achieve the goals advocated for in the WHO’s HBHI strategy [51]. Although not undertaken as part of this study, additional research by NMCPs on the costs and cost-effectiveness would be valuable to estimate the financial implications of combining IRS and LLINs long-term to not only reduce morbidity and mortality but also to decrease the size of the reservoir of infection. Such analyses are fiscally prudent with the shift towards use of more expensive insecticides [1]. Given the persistence of this substantial and diverse *P*. *falciparum* reservoir in residents of all ages and its role in sustaining onward transmission [52, 53], rebound is a likely outcome should IRS be discontinued. For IRS to be successful in high-transmission areas like Bongo it must be sustained, ideally in combination with LLINs and targeted chemotherapy (e.g., mass drug administration), to address the reservoir of infection in all ages.

## Supporting information

S1 Checklist Inclusively in global research. (PDF)

S1 Fig The proportion microscopic *P*. *falciparum* infections categorized into low (40–999 parasites/μL), moderate (1,000–9,999 parasites/μL), and high (≥ 10,000 parasites/μL parasites/μL) parasite densities groups pre- to post-IRS at the end of the wet (blue) and dry (gold) seasons. Proportion of microscopic *P*. *falciparum* infections categorized pre- to post-IRS at the **A.** end of the wet and dry season surveys and **B.** across each age group (years) at the end of the wet and dry season surveys.
(TIF)

S2 Fig Density of microscopic *P*. *falciparum* infections grouped based on anaemia status pre- to post-IRS at the end of the wet (blue) and dry (gold) seasons. Box and whisker plots of the log-transformed microscopic *P*. *falciparum* infection densities (parasites/μL) grouped based on anaemia status pre- to post-IRS **A.** at the end of the wet season surveys and **B.** at the end of the dry season surveys. The boxes represent the inter-quartile ranges (IQR) and the horizontal lines represent the median log_10_-transformed *P*. *falciparum* infection densities (parasites/μL). The whiskers are used to depict the largest and smallest log_10_-transformed infection densities and the grey dots outside the whiskers are used to denote outliers. The parasite densities were log_10_-transformed to remove skewness. Anaemia status was defined according to the WHO guidelines for age and gender (S1 Table). (Median *P*. *falciparum* density (value/μL), Inter Quartile Range [IQR] in anaemic vs. non-anaemic: Survey 1 (640 [200 – 2,800] vs. 400 [160–1,080], respectively), Survey 2 (320 [120–1,30] vs. 120 [80–360], respectively), Survey 3 (520 [120–2,690] vs. 240 [80–1,280], respectively), Survey 4 (320 [120–710] vs. 250 [120–560], respectively).
(TIF)

S3 Fig The proportion of microscopic *P*. *falciparum* infections categorized as single-genome (estimated MOI_*var*_ = 1) or multi-genome (estimated MOI_*var*_ > 1) pre- to post-IRS at the end of the wet (blue) and dry (gold) seasons. Proportion of microscopic *P*. *falciparum* infections categorized pre- to post-IRS at the **A.** end of the wet and dry season surveys and **B.** across each age group (years) at the end of the wet and dry season surveys.
(TIF)

S4 Fig Relative MOI_*var*_ frequency distributions pre- to post-IRS at the end of the wet (blue) and dry (gold) seasons. On the horizontal axis are the discrete estimated MOI_*var*_ categories (i.e., range MOI_*var*_ 1–20) for each microscopic *P*. *falciparum* infection. The vertical axis depicts the relative proportion of infections found in each of the these MOI_*var*_ categories at the **A.** end of the wet and dry season surveys and **B.** across each age group (years) at the end of the wet and dry season surveys. For additional details on the number of *P*. *falciparum* infections during each survey see S4 Table.
(TIF)

S1 Table Demographic characteristics of the study population during each survey. (PDF)

S2 Table Parasitological parameters and DBLα type sequencing data for the microscopic *P*. *falciparum* infections during each survey. (PDF)

S3 Table DBLα type sequencing results for all isolates that were positive for a *P*. *falciparum* infection by microscopy. (PDF)

S4 Table Parasitological parameters of the *P*. *falciparum* infections during each survey. (PDF)

S5 Table Absolute decrease in the probability of having a microscopic *P*. *falciparum* infection post-IRS in Bongo at the end of the wet and dry seasons. Results are expressed in terms of Attributable Risk (AR) and Attributable Risk percentage (AR%).
(PDF)

S6 Table Association between the IRS and microscopic *P*. *falciparum* infection prevalence at the end of the wet seasons. (PDF)

S7 Table Association between the IRS and microscopic *P*. *falciparum* infection prevalence at the end of the dry seasons. (PDF)

S8 Table Stratum-specific estimates for the association between the IRS and microscopic *P*. *falciparum* prevalence at the end of the wet seasons. The reference for all comparisons was Survey 1 (pre-IRS, October 2012).
(PDF)

S9 Table Stratum-specific estimates for the association between the IRS and microscopic *P*. *falciparum* infection prevalence at the end of the dry seasons. The reference for all comparisons was Survey 2 (pre-IRS, May/June 2013).
(PDF)

S10 Table Association between the IRS and *P*. *falciparum* infection (i.e., microscopic or submicroscopic) prevalence at the end of the wet seasons. (PDF)

S11 Table Absolute decrease in the probability of having a *P*. *falciparum* infection (i.e., microscopic or submicroscopic) post-IRS in Bongo at the end of the wet season. Results are expressed in terms of Attributable Risk (AR) and Attributable Risk percentage (AR%).
(PDF)

S12 Table Stratum-specific estimates for the association between the IRS and *P*. *falciparum* (i.e., microscopic or submicroscopic) infection prevalence at the end of the wet seasons. The reference for all comparisons was Survey 1 (pre-IRS, October 2012).
(PDF)

S13 Table The estimated number of *P*. *falciparum* genomes. (PDF)

S1 Text Structured questionnaire. (PDF)
