# Supplementary material for: Indoor residual spraying with a non-pyrethroid insecticide reduces the reservoir of Plasmodium falciparum in a high-transmission area in northern Ghana
Source: PLOS Glob Public Health. 2022 May 18;2(5):e0000285. doi: 10.1371/journal.pgph.0000285 (PMC9121889; doi:10.1371/journal.pgph.0000285)
Supplement: S1 Checklist — (PDF) [file pgph.0000285.s001.pdf]

S1 Checklist. Inclusively in global research

Indoor residual spraying with a non-pyrethroid insecticide reduces the reservoir of *Plasmodium falciparum* in a high-transmission area in northern Ghana

Kathryn E. Tiedje[¶], Abraham R. Oduro[¶], Oscar Bangre, Lucas Amenga-Etego, Samuel K Dadzie, Maxwell A Appawu, Kwadwo Frempong, Victor Asoala, Shazia Ruybal-Pésantez, Charles A. Narh, Samantha L. Deed, Dionne C. Argyropoulos, Anita Ghansah, Samuel A. Agyei, Sylvester Segbaya, Kwame Desewu, Ignatius Williams, Julie A. Simpson, Keziah Malm, Mercedes Pascual, Kwadwo A. Koram, and Karen P. Day*

¶ KET and ARO contributed equally to this work as first authors.

* Corresponding author: Karen P. Day; karen.day@unimelb.edu.au

Inclusivity in global research

PLOS' policy on inclusivity in global research aims to improve transparency in the reporting of research performed outside of researchers' own country or community and ensures that PLOS publications reporting global research adhere to high standards for research ethics and authorship. Authors of relevant research articles may be asked to complete the questionnaire below, which outlines ethical, cultural, and scientific considerations specific to inclusivity in global research. This questionnaire may be requested when researchers have travelled to a different country to conduct research, if research uses samples collected in another country, research with Indigenous populations or their lands, or if research is on cultural artefacts. Researchers travelling to another country solely to use laboratory equipment will not normally be required to complete the questionnaire. However, the questionnaire can be requested at the journal's discretion for any submission – if you have been requested to complete this questionnaire by the PLOS journal you submitted to, please do so.

Please complete the questionnaire below and include this as a Supporting Information file with your manuscript. Note that if your paper is accepted for publication, this checklist will be published with your article in the supporting information files. Please ensure that you reference the checklist in the main body of your manuscript. We suggest adding a subsection 'Inclusivity in global research' to your Methods section and adding the following sentence: "Additional information regarding the ethical, cultural, and scientific considerations specific to inclusivity in global research is included in the Supporting Information (SX Checklist)"

The questions have been designed to be applicable to a wide range of study types, and there are subsections for both human subjects research and non-human subjects research. If any of the questions are not relevant to your research please mark them as "N/A" as appropriate.

Ethical considerations, permits and authorship

This section is applicable to all research types.

Provide details as to who granted permissions and/or consent for the study to take place in the Methods section of your manuscript. This should include the names of **all** ethics boards, governmental organizations, community leaders or other bodies that provided approval for the study. If individuals provided approval refer to these people by their role or title but do not list their name(s).

Reported on page number: Reported on page number: 16 of the Methods and Materials in the "Ethical approval" section.

If there were any deviations from the study protocol after approval was obtained please provide details of these changes in the Methods section of your manuscript.

Reported on page number: N/A

Did this study involve local collaborators that are residents of the country where the research was conducted or members of the community studied? If you do not have any authors from said communities, please provide an explanation for this below.

Yes, this study involved local collaborators that are residents of Ghana and/or members of the Bongo District community being studied. The manuscript was co-first authored by Abraham R. Oduro who is from Navrongo Health Research Centre (Navrongo, Upper East Region, Ghana) and is based in Ghana. In addition, other co-authors from Ghana were directly involved with this study: Oscar Bangre, Lucas Amenga-Etego, Samuel K Dadzie, Maxwell A Appawu, Kwadwo Frempong, Victor Asoala, Charles A. Narh, Anita Ghansah, Samuel A. Agyei, Sylvester Segbaya, Kwame Desewu, Ignatius Williams, Keziah Malm, and Kwadwo A. Koram.

Everyone listed as an author should meet PLOS' criteria for authorship and all individuals who meet these criteria should be included in the author byline, rather than the acknowledgements. Authorship criteria is based on the International Committee of Medical Journal Editors (ICMJE) Uniform Requirements for Manuscripts Submitted to Biomedical Journals - for further information please see here: https://journals.plos.org/plosone/s/authorship.

Human subjects research (e.g. health research, medical research, cross-cultural psychology)

Did you obtain written informed consent from a representative of the local community or region before the research took place? How did you establish who speaks for the community? Details of written informed consent obtained from study participants should be reported separately in the Methods section of your manuscript.

Yes, prior to this research taking place, written informed consent was sought and obtained from the key stakeholders in Bongo District, specifically the Paramount Chief of the Bongo traditional area, the Bongo District Assembly, the Bongo District Health Directorate, and the Regional Health Directorate (Upper East Region). To engage with the local Bongo District community prior to the start of the study, community durbars were organized with the traditional rulers and community members. Community consent was granted to the investigators to proceed with the study. The study coordinator(s) from the Navrongo Health Research Center directed and presented at these key stakeholder meetings and local community durbars in Bongo District. They study coordinator(s) were specifically chosen as they were from the region, understand the local customs, and spoke the local language (i.e., Gurene).

How did members of the local community provide input on the aims of the research investigation, its methodology, and its anticipated outcome(s)?

The members of the local community provided a clear definition of the boundaries for agroecological zones (i.e., catchment areas Vea/Gowrie and Soe), community profiles/details, and population structure at the baseline. This information helped the research team to refocus some of the study objectives.

When engaging with the local community, how did you ensure that the informed consent documents and other materials could be understood by local stakeholders?

All informed consent documents (i.e., consent/assent forms, plain language states, oral consent procedures/text) were presented to the local stakeholders by the study coordinator(s) from the Navrongo Health Research Center. These meetings involved presentations and discussions on these documents, which were prepared and provided in the local language (i.e., Gurene) and English. During these meetings the local stakeholders also had a chance to speak directly to the study coordinator(s) if they required more information and/or had any questions. In addition, prior to the start of the study in Bongo District, sensitization meetings were organized to the explain the purpose of the study to the local community and to provide an opportunity for community members to ask questions, provide comments, etc.

Will the findings of the research be made available in an understandable format to stakeholders in the community where the study was conducted (e.g. via a presentation, summary report, copies of publications, etc.)? Please provide details of how this will be achieved.

The findings of this research have been made available to the key stakeholders and local community (i.e., Paramount Chief of Bongo, his divisional chiefs, the Queen Mothers, and community members) using community-based debriefing presentations/meetings and radio broadcasts. In addition, presentations/meetings were also undertaken with the Bongo District Health Directorate and the Regional Health Directorate (Upper East Region). These presentations/meetings were prepared, coordinated, and delivered by the study coordinator(s) from the Navrongo Health Research Center in the local language (i.e., Gurene) and English, so that they were accessible to the local community. During these presentations/meetings, the study coordinator(s) were available to respond to questions and comments. Finally, summary reports and copies of publications related to this research have been made and will continue to be made available to key stakeholders and local community.

Non-human subjects research using specimens/ animals collected as part of the study, or those housed in archival collections. Examples include archaeology, paleontology, botany and zoology.

Did the permission you obtained from a local authority to perform the study include an agreement on access to outputs and benefit sharing? This may include procedures to enable fair distribution of the benefits and resources arising from the research performed. Please include any details of Prior Informed Consent and Benefit Sharing Agreements obtained. These may be required by field-specific regulations, for example the Convention on Biological Diversity (CBD) and the associated Nagoya Protocol.

Permission was obtained from the independent Ethics Bodies/Institutional Boards in Ghana who operate according to ICH/GCP principles and ensure there is equitable/fair distribution of resources and that the research will have benefits for the local community. The research team and the funding agencies are bound by ethical principles to ensure that the informed consent documents clearly defined the risks and benefits of being involved in this study.

If the material used in your study was imported, please A) provide the year it was imported and B) indicate whether permits were obtained to import/export the materials used, C) provide details of any permits obtained. If this information is not available, please indicate this.

A) The biological specimens (i.e., dried blood spots) for this study were imported into Australia between 2014 and 2016.
B) All necessary import permits were obtained to import of these biological specimens (i.e., dried blood spots) into Australia. No export permits were required for these shipments to be sent from Ghana.
C) These three shipments were covered by two different import permits issued to the University Melbourne by the Australian government (AIQS-IP14004415 and AIQS-0000356592). A Material Transfer Agreement for the shipment of the biological samples from Navrongo Health Research Centre to University of Melbourne was signed prior to the shipments.

If you used archival specimens, please state how the material used in your study was acquired by the institute it is held in and provide details of any permits obtained for the original excavations/ sample collection. If this information is not available, please indicate this.

N/A

How was the potential cultural significance of the materials collected in your study to local communities considered in your research design? Were Indigenous peoples and/or local researchers and institutions involved with archaeological excavations / collection of specimens? If so, please provide a description of their involvement.

All biological specimens (i.e., dried blood spots) collected in this study were collected in Bongo District by local researchers from the Navrongo Health Research Center. In addition, literate local residents (Gurene and English) from Bongo District were trained as field workers and were directly involved in liaising with the local community and in the collection of study data onto the structured questionnaires.

If your manuscript includes photographs of human remains please indicate whether authors obtained permission from descendants or affiliated cultural communities to do so.

N/A
