# Supplementary figures and images for: Indoor residual spraying with a non-pyrethroid insecticide reduces the reservoir of Plasmodium falciparum in a high-transmission area in northern Ghana
Source: PLOS Glob Public Health. 2022 May 18;2(5):e0000285. doi: 10.1371/journal.pgph.0000285 (PMC9121889; doi:10.1371/journal.pgph.0000285)

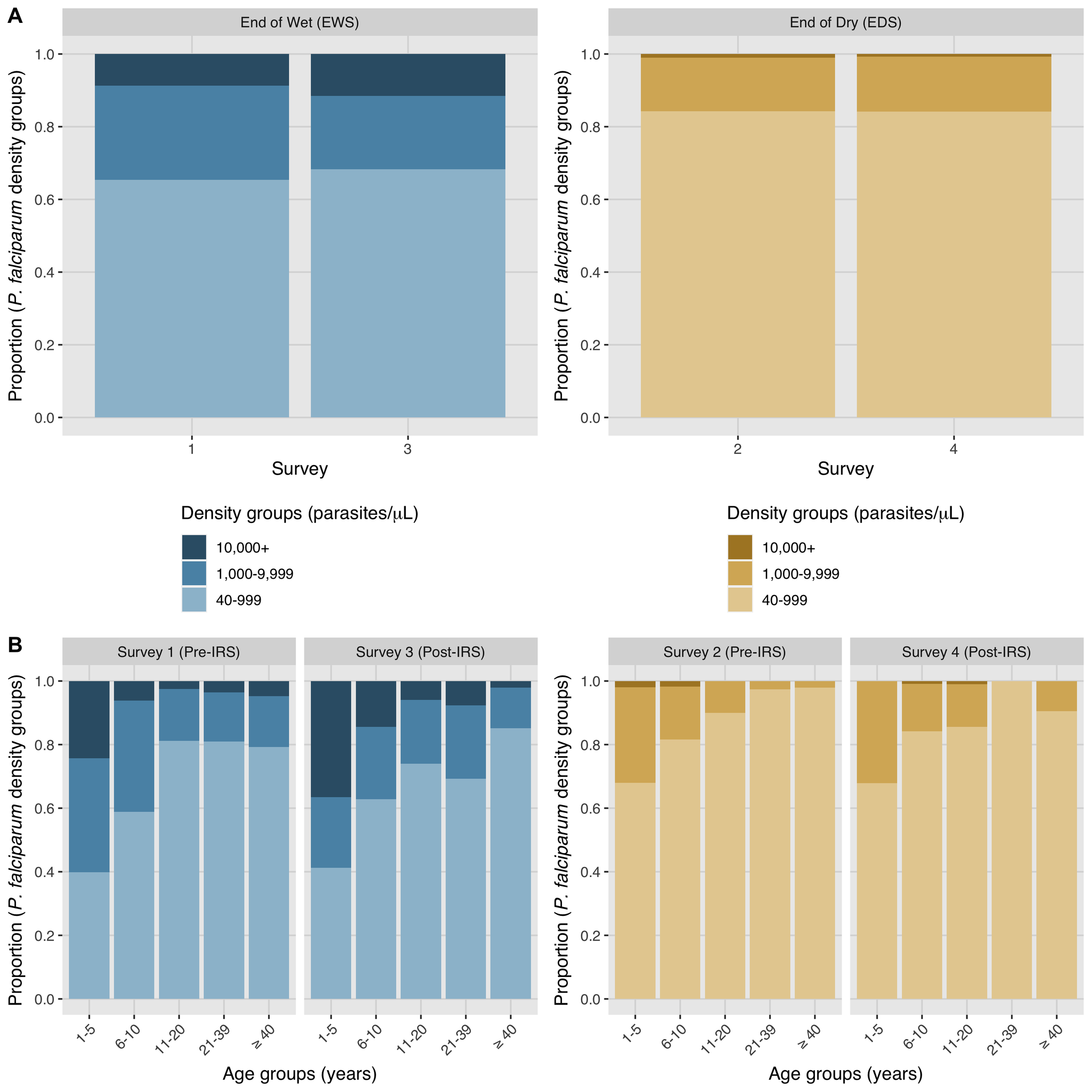

Supplement: S1 Fig — Proportion of microscopic P. falciparum infections categorized pre- to post-IRS at the A. end of the wet and dry season surveys and B. across each age group (years) at the end of the wet and dry season surveys. (TIF) [file pgph.0000285.s002.tif]

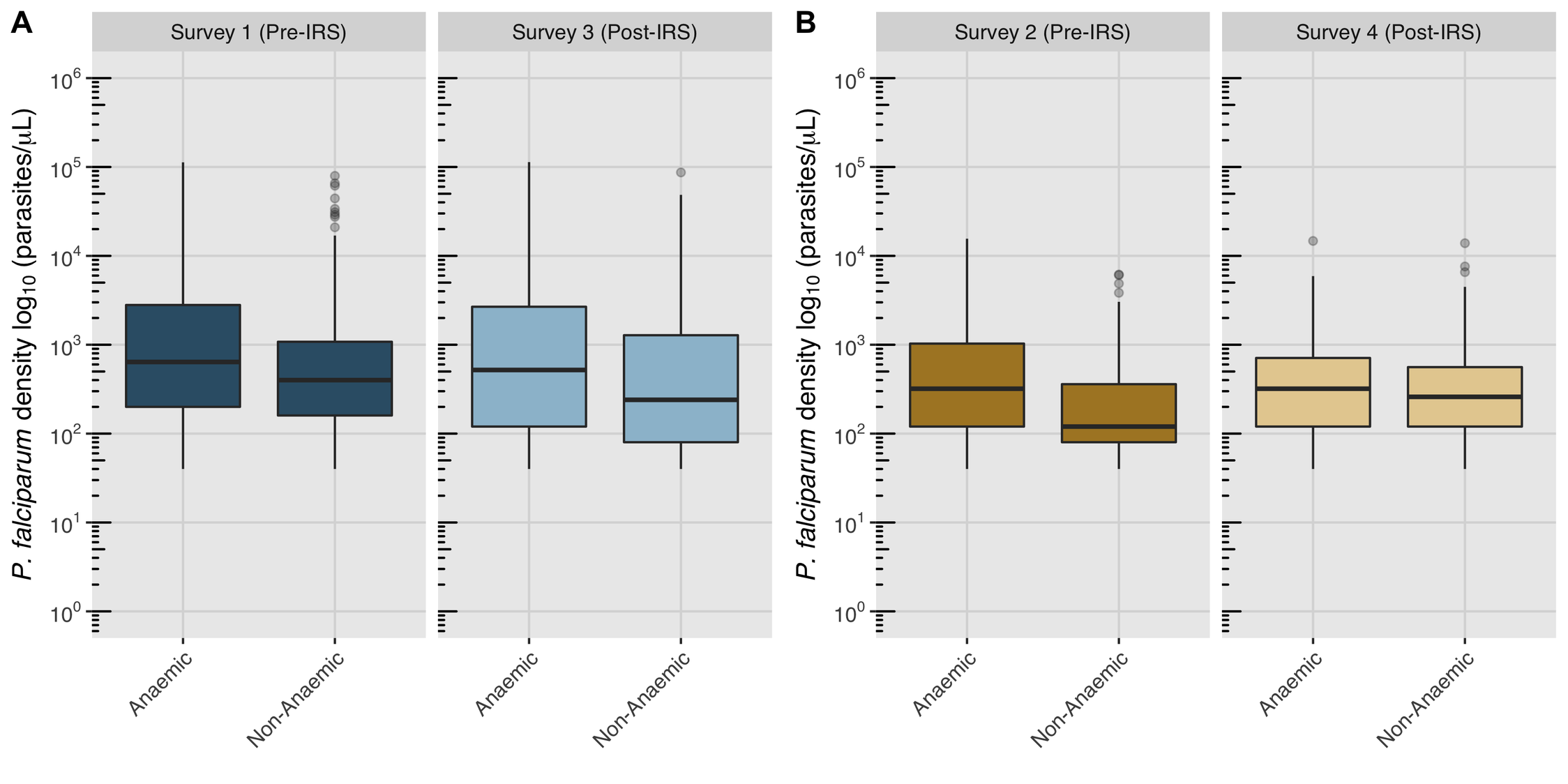

Supplement: S2 Fig — Box and whisker plots of the log-transformed microscopic P. falciparum infection densities (parasites/μL) grouped based on anaemia status pre- to post-IRS A. at the end of the wet season surveys and B. at the end of the dry season surveys. The boxes represent the inter-quartile ranges (IQR) and the horizontal lines represent the median log10-transformed P. falciparum infection densities (parasites/μL). The whiskers are used to depict the largest and smallest log10-transformed infection densities and the grey dots outside the whiskers are used to denote outliers. The parasite densities were log10-transformed to remove skewness. Anaemia status was defined according to the WHO guidelines for age and gender (S1 Table). (Median P. falciparum density (value/μL), Inter Quartile Range [IQR] in anaemic vs. non-anaemic: Survey 1 (640 [200 – 2,800] vs. 400 [160–1,080], respectively), Survey 2 (320 [120–1,30] vs. 120 [80–360], respectively), Survey 3 (520 [120–2,690] vs. 240 [80–1,280], respectively), Survey 4 (320 [120–710] vs. 250 [120–560], respectively). (TIF) [file pgph.0000285.s003.tif]

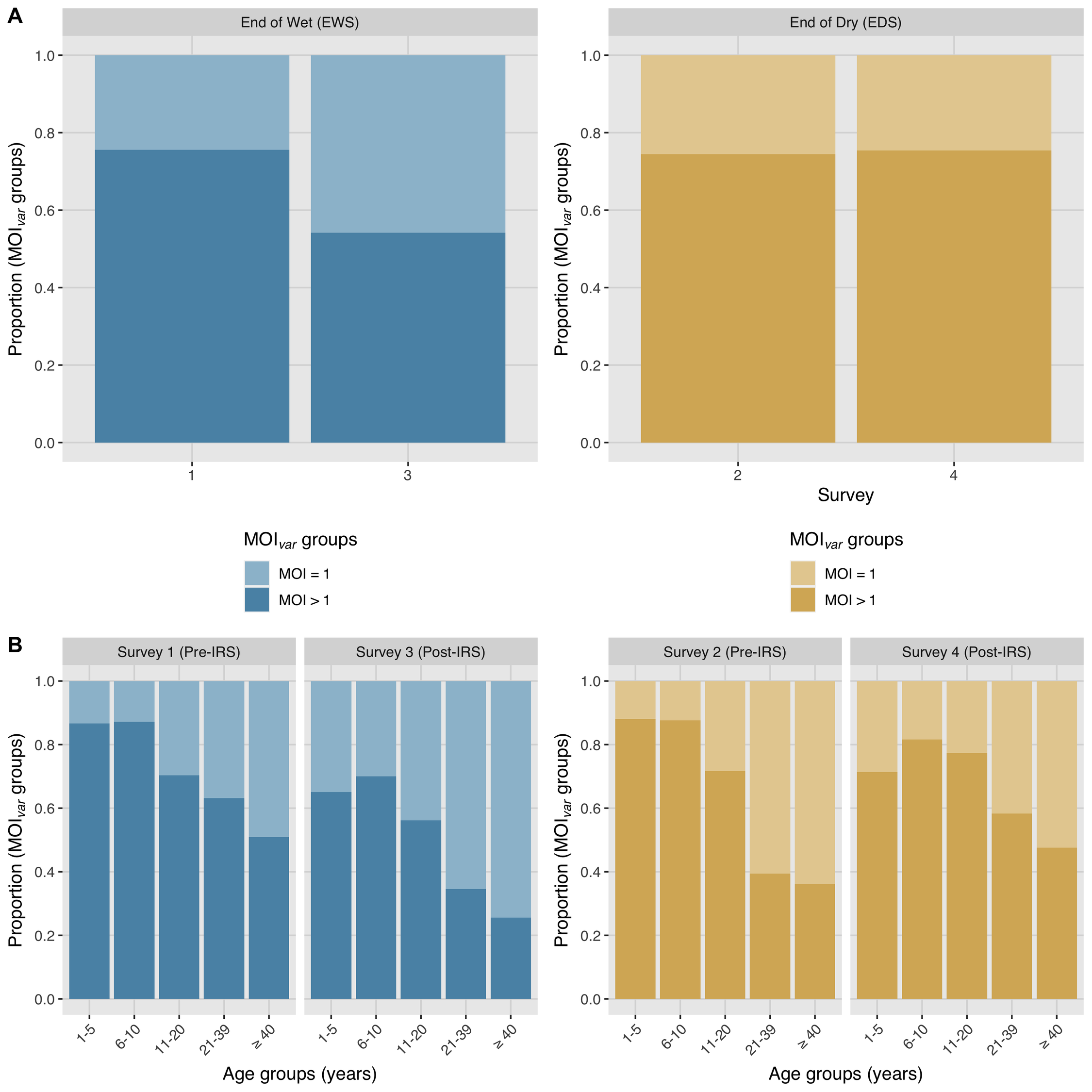

Supplement: S3 Fig — Proportion of microscopic P. falciparum infections categorized pre- to post-IRS at the A. end of the wet and dry season surveys and B. across each age group (years) at the end of the wet and dry season surveys. (TIF) [file pgph.0000285.s004.tif]

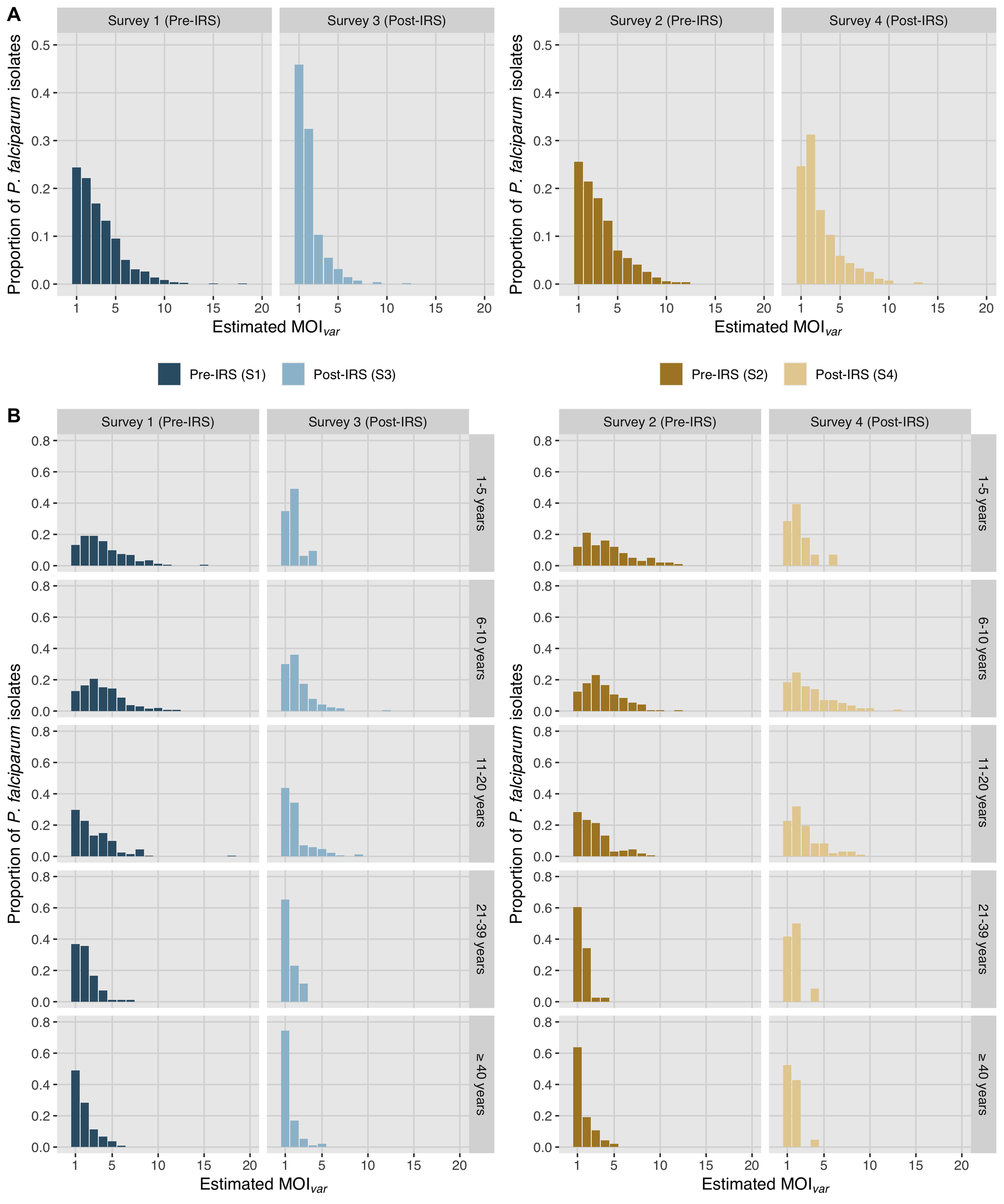

Supplement: S4 Fig — On the horizontal axis are the discrete estimated MOIvar categories (i.e., range MOIvar 1–20) for each microscopic P. falciparum infection. The vertical axis depicts the relative proportion of infections found in each of the these MOIvar categories at the A. end of the wet and dry season surveys and B. across each age group (years) at the end of the wet and dry season surveys. For additional details on the number of P. falciparum infections during each survey see S4 Table. (TIF) [file pgph.0000285.s005.tif]
