# Supplementary material for: Indoor residual spraying with a non-pyrethroid insecticide reduces the reservoir of Plasmodium falciparum in a high-transmission area in northern Ghana
Source: PLOS Glob Public Health. 2022 May 18;2(5):e0000285. doi: 10.1371/journal.pgph.0000285 (PMC9121889; doi:10.1371/journal.pgph.0000285)
Supplement: S1 Table — (PDF) [file pgph.0000285.s006.pdf]

S1 Table. Demographic characteristics of the study population during each survey.

Demographic characteristics [a]	Pre-IRS		Post-IRS	
	Survey 1 End of wet season (October 2012)	Survey 2 End of dry season (May/June 2013)	Survey 3 End of wet season (October 2015)	Survey 4 End of dry season (May/June 2016)
Age groups [b]				
All	1923	1902	2022	2091
1-5 years	356 (18.5)	351 (18.5)	405 (20.0)	358 (17.1)
6-10 years	395 (20.5)	404 (21.2)	409 (20.2)	425 (20.3)
11-20 years	413 (21.5)	406 (21.3)	467 (23.1)	514 (24.6)
21-39 years	326 (17.0)	315 (16.6)	297 (14.7)	331 (15.8)
≥ 40 years	433 (22.5)	426 (22.4)	444 (22.0)	463 (22.2)
Sex [c]				
Female	1031 (53.6)	1055 (55.5)	1093 (54.1)	1124 (53.8)
Male	892 (46.4)	847 (44.5)	929 (45.9)	967 (46.2)
Catchment area [d]				
Vea/Gowrie	919 (47.8)	925 (46.8)	1000 (49.5)	1026 (49.1)
Soe	1004 (52.2)	977 (51.4)	1022 (50.5)	1065 (50.9)
LLIN usage (previous night)				
No	210 (10.9)	286 (15.0)	191 (9.4)	274 (13.1)
Yes	1713 (89.1)	1616 (85.0)	1831 (90.6)	1817 (86.9)
Antimalarial treatment (previous 2-weeks) [e]				
No treatment	1127 (58.6)	1748 (91.9)	1645(81.4)	1922 (91.9)
Treatment	796 (41.4)	154 (8.1)	298 (14.7)	157 (7.6)
Don't know	0 (0)	0 (0)	79 (3.9)	12 (0.6)
Anaemia Status [f]				
Anaemic	896 (46.7)	605 (32.2)	829 (41.1)	602 (28.8)
Non-anaemic	1,022 (53.3)	1,275 (67.8)	1,188 (58.9)	1,487 (71.2)

[a] Data reflect the number (% (n/N)) of subjects.
[b] During each survey a similar proportion of participants were surveyed in all the age groups.
[c] During each survey a similar proportion of female and male participants were surveyed in each age category, except for the adult age groups (>20 years) where significantly more females than males were surveyed (p-value ≤ 0.038).
[d] During each survey a similar proportion of participants were surveyed in each catchment area.
[e] Indicates those participants who reported they were sick, sought treatment, and were provided with an antimalarial treatment in the previous two-weeks.
[f] Anaemia status was defined according to the WHO guidelines for age and gender. Participants in each Survey were excluded from the anaemia status category if their haemoglobin was not measured on the day the survey was conducted: Survey 1 (N = 5), Survey 2 (N = 22), Survey 3 (N = 5), and Survey 4 (N = 2).
