# Supplementary material for: Indoor residual spraying with a non-pyrethroid insecticide reduces the reservoir of Plasmodium falciparum in a high-transmission area in northern Ghana
Source: PLOS Glob Public Health. 2022 May 18;2(5):e0000285. doi: 10.1371/journal.pgph.0000285 (PMC9121889; doi:10.1371/journal.pgph.0000285)
Supplement: S2 Table — (PDF) [file pgph.0000285.s007.pdf]

S2 Table. Parasitological parameters and DBLα type sequencing data for the microscopic *P. falciparum* infections during each survey.

Parasitological parameters	Pre-IRS		Post-IRS	
	Survey 1<br>End of wet season<br>(October 2012)	Survey 2<br>End of dry season<br>(May/June 2013)	Survey 3<br>End of wet season<br>(October 2015)	Survey 4<br>End of dry season<br>(May/June 2016)
Number of participants [a]	1923 (100)	1902 (100)	2022 (100)	2091 (100)
Microscopic *P. falciparum* prevalence [b]	808 (42.0)	513 (27.0)	545 (27.0)	272 (13.0)
Age groups				
1-5 years	173 (48.6)	100 (28.5)	63 (15.6)	28 (7.8)
6-10 years	243 (61.5)	169 (41.8)	167 (40.8)	114 (26.5)
11-20 years	202 (48.9)	159 (39.2)	169 (36.2)	97 (18.9)
>20 years	190 (25.0)	85 (11.5)	146 (19.7)	33 (4.2)
Microscopic *P. falciparum* isolates with DBLα type sequencing data [c]	685 (84.8)	440 (85.8)	413 (75.8)	238 (87.5)
Age groups				
1-5 years	158 (91.3)	94 (94.0)	51 (81.0)	25 (89.3)
6-10 years	217 (89.3)	156 (92.3)	146 (87.4)	103 (90.4)
11-20 years	167 (82.7)	136 (85.5)	129 (76.3)	85 (87.6)
>20 years	143 (75.3)	54 (63.5)	87 (59.6)	25 (75.8)

[a] Number of participants surveyed that were analysed by microscopy.

[b] Data reflect the number (% (n/N)) of participants sampled that were microscopically positive for *P. falciparum* (including mixed *P. falciparum* infections) relative to the number of participants surveyed in the total population or by the age groups presented.

[c] Data reflect the number (% (n/N)) of microscopic *P. falciparum* isolates that had DBLα type sequencing data relative the number of participants surveyed that were microscopically positive for *P. falciparum* (including mixed *P. falciparum* infections) in the total population or by the age groups presented. *P. falciparum* isolates that had low DNA quality and/or sequencing quality (i.e., < 20 DBLα types, S3 Table) were removed when the sequencing dataset was cleaned (please see the Methods for additional details).
