# Supplementary material for: Indoor residual spraying with a non-pyrethroid insecticide reduces the reservoir of Plasmodium falciparum in a high-transmission area in northern Ghana
Source: PLOS Glob Public Health. 2022 May 18;2(5):e0000285. doi: 10.1371/journal.pgph.0000285 (PMC9121889; doi:10.1371/journal.pgph.0000285)
Supplement: S3 Table — (PDF) [file pgph.0000285.s008.pdf]

S3 Table. DBLα type sequencing results for all isolates that were positive for a *P. falciparum* infection by microscopy.

Survey	*P. falciparum* positive isolates sequenced	*P. falciparum* isolates with DBLα type sequencing data (i.e., ≥ 20 DBLα types) [a]	*P. falciparum* isolates with no DBLα type sequencing data (i.e., 0 DBLα types) [b]	*P. falciparum* isolates with limited DBLα type sequencing data (i.e., 1 to < 20 DBLα types) [c]
1	808	685 (84.8)	66 (8.2)	57 (7.0)
2	513	440 (85.8)	45 (8.8)	28 (5.4)
3	545	413 (75.8)	35 (6.4)	97 (17.8)
4	272	238 (87.5)	13 (4.8)	21 (7.7)
TOTAL	2,138	1,776 (83.1)	159 (7.4)	203 (9.5)

[a] Data reflect the number (% (n/N)) of *P. falciparum* isolates that had DBLα type sequencing data relative to the number of participants sampled that had microscopically positive for *P. falciparum* (including mixed *P. falciparum* infections).

[b] Data reflect the number (% (n/N)) of microscopic *P. falciparum* isolates that had no DBLα type sequencing data relative to the number of participants sampled that were microscopically positive for *P. falciparum* (including mixed *P. falciparum* infections).

[c] Data reflect the number (% (n/N)) of microscopic *P. falciparum* isolates that had between DBLα type sequencing data (i.e., 1 to <20 DBLα types) relative the number of participants sampled that were microscopically positive for *P. falciparum* (including mixed *P. falciparum* infections).
