# Supplementary material for: Indoor residual spraying with a non-pyrethroid insecticide reduces the reservoir of Plasmodium falciparum in a high-transmission area in northern Ghana
Source: PLOS Glob Public Health. 2022 May 18;2(5):e0000285. doi: 10.1371/journal.pgph.0000285 (PMC9121889; doi:10.1371/journal.pgph.0000285)
Supplement: S4 Table — (PDF) [file pgph.0000285.s009.pdf]

S4 Table. Parasitological parameters of the *P. falciparum* infections during each survey.

Parasitological parameters	Pre-IRS		Post-IRS	
	Survey 1<br>End of wet season<br>(October 2012)	Survey 2<br>End of dry season<br>(May/June 2013)	Survey 3<br>End of wet season<br>(October 2015)	Survey 4<br>End of dry season<br>(May/June 2016)
Number of participants [a]	1923 (100)	1902 (100)	2022 (100)	2091 (100)
Microscopic *P. falciparum* prevalence [b]				
Age groups				
All	**808 (42.0)**	**513 (27.0)**	**545 (27.0)**	**272 (13.0)**
1-5 years	173 (48.6)	100 (28.5)	63 (15.6)	28 (7.8)
6-10 years	243 (61.5)	169 (41.8)	167 (40.8)	114 (26.5)
11-20 years	202 (48.9)	159 (39.2)	169 (36.2)	97 (18.9)
21-39 years	84 (25.8)	38 (12.1)	52 (17.5)	12 (3.6)
≥ 40 years	106 (24.5)	47 (11.0)	94 (21.2)	21 (4.5)
Sex				
Female	379 (36.8)	239 (22.6)	269 (24.6)	123 (10.9)
Male	429 (48.1)	274 (32.3)	276 (29.7)	149 (15.4)
Catchment area				
Vea/Gowrie	356 (38.7)	255 (27.6)	261 (26.1)	97 (9.5)
Soe	452 (45.0)	258 (26.4)	284 (27.8)	175 (16.4)
Microscopic *P. falciparum* density [c]				
Age groups				
All	**520 [160-1640]**	**160 [80-560]**	**320 [120-1800]**	**280 [120-640]**
1-5 years	1640 [400-9840]	440 [120-1270]	1840 [240-19,940]	400 [80-1650]
6-10 years	760 [240-1840]	240 [120-680]	520 [200-2720]	380 [160-720]
11-20 years	320 [160-760]	120 [80-400]	280 [120-1000]	240 [160-480]
21-39 years	200 [120-720]	120 [40-160]	200 [80-1730]	120 [80-200]
≥ 40 years	200 [120-670]	80 [40-140]	120 [40-320]	120 [80-160]
Sex				
Female	520 [160-1620]	160 [80-460]	320 [80-1960]	280 [120-480]
Male	480 [160-1640]	200 [120-600]	320 [120-1300]	320 [120-720]
Catchment area				
Vea/Gowrie	360 [200-1200]	160 [80-520]	240 [80-1280]	280 [120-560]
Soe	680 [200-2170]	200 [80-600]	400 [120-2210]	280 [120-640]
Submicroscopic *P. falciparum* prevalence [d,e]	1115 (100)		1473 (100)	
Age groups				
All	**612 (54.9)**		**295 (20.0)**	
1-5 years	82 (44.8)		36 (11.1)	
6-10 years	90 (59.2)		56 (23.1)	
11-20 years	145 (68.7)		93 (31.4)	
21-39 years	128 (52.9)		51 (20.9)	
≥ 40 years	167 (51.1)		57 (16.3)	
Sex				
Female	340 (52.1)		149 (18.1)	
Male	272 (58.7)		146 (22.5)	
Catchment area				
Vea/Gowrie	258 (45.1)		118 (16.0)	
Soe	354 (64.1)		177 (24.0)	

[a] Number of participants surveyed that were analysed by microscopy.
[b] Data reflect the number (% (n/N)) of participants that were microscopically positive for *P. falciparum* (including mixed *P. falciparum* infections).
[c] Median parasite density for microscopically positive *P. falciparum* (including mixed *P. falciparum* infections) (value/µL, Inter Quartile Range [IQR]) samples.
[d] Data reflect the number (% (n/N)) of participants sampled in Survey 1 and Survey 3 that were PCR positive (i.e., submicroscopic) for *P. falciparum* (including mixed *P. falciparum* infections).
[e] For the submicroscopic *P. falciparum* infections in Survey 3, there were 1,473 participants included in the analysis: exclusions were participants where a dried blood spot was not available for PCR (N=4).
