# Supplementary material for: Indoor residual spraying with a non-pyrethroid insecticide reduces the reservoir of Plasmodium falciparum in a high-transmission area in northern Ghana
Source: PLOS Glob Public Health. 2022 May 18;2(5):e0000285. doi: 10.1371/journal.pgph.0000285 (PMC9121889; doi:10.1371/journal.pgph.0000285)
Supplement: S5 Table — Results are expressed in terms of Attributable Risk (AR) and Attributable Risk percentage (AR%). (PDF) [file pgph.0000285.s010.pdf]

S5 Table. Absolute decrease in the probability of having a microscopic *P. falciparum* infection post-IRS in Bongo at the end of the wet and dry seasons. Results are expressed in terms of Attributable Risk (AR) and Attributable Risk percentage (AR%).

**Outcome**	**Demographic characteristics**	**Pre-IRS to Post-IRS End of wet season Survey 1 to Survey 3**			**Pre-IRS to Post-IRS End of dry season Survey 2 to Survey 4**		
		**AR (95% CI)**	**AR% (95% CI)**	***p*-value**	**AR (95% CI)**	**AR% (95% CI)**	***p*-value**
Positive for a microscopic *P. falciparum* infection	**All**	0.151 (0.121, 0.180)	36.9 (29.9, 41.3)	< 0.001	0.140 (0.115, 0.164)	51.8 (44.9, 57.8)	< 0.001
	**Age Groups**						
	1-5 years	0.330 (0.268, 0.393)	68.0 (58.9, 75.1)	< 0.001	0.207 (0.152, 0.262)	72.6 (59.4, 81.5)	< 0.001
	6-10 years	0.207 (0.139, 0.275)	33.6 (23.6, 42.3)	< 0.001	0.150 (0.086-0.214)	35.9 (22.1, 47.2)	< 0.001
	11-20 years	0.127 (0.062, 0.192)	26.0 (13.6, 36.7)	< 0.001	0.203 (0.145, 0.261)	51.8 (40.2, 61.2)	< 0.001
	21-39 years	0.083 (0.018, 0.147)	32.1 (7.5, 50.1)	0.013	0.084 (0.043, 0.126)	70.0 (43.6, 84.0)	0.013
	≥ 40 years	0.033 (-0.022, 0.089)	13.5 (-10.4, 32.3)	0.243	0.065 (0.030, 0.100)	58.9 (32.4, 75.0)	0.243
	**Sex**						
	Female	0.122 (0.083, 0.161)	33.1 (23.6, 41.3)	< 0.001	0.117 (0.086, 0.148)	51.7 (41.0, 60.5)	< 0.001
	Male	0.184 (0.140, 0.228)	38.2 (30.3, 45.2)	< 0.001	0.169 (0.131, 0.208)	52.4 (43.2, 60.1)	< 0.001
	**Catchment area**						
	Vea/Gowrie	0.126 (0.085, 0.168)	32.6 (23.1, 41.0)	< 0.001	0.181 (0.147, 0.215)	65.7 (57.4, 72.4)	< 0.001
	Soe	0.172 (0.131, 0.214)	38.3 (30.4, 45.3)	< 0.001	0.100 (0.064, 0.135)	37.8 (26.2, 47.6)	< 0.001

AR (Attributable Risk = (Pre-IRS Risk) - (Post-IRS Risk))
AR% (Attributable Risk Percentage = ((Pre-IRS Risk) - (Post-IRS Risk)/ (Pre-IRS Risk)) * 100%)
CI=confidence interval
