# Supplementary material for: Indoor residual spraying with a non-pyrethroid insecticide reduces the reservoir of Plasmodium falciparum in a high-transmission area in northern Ghana
Source: PLOS Glob Public Health. 2022 May 18;2(5):e0000285. doi: 10.1371/journal.pgph.0000285 (PMC9121889; doi:10.1371/journal.pgph.0000285)
Supplement: S6 Table — (PDF) [file pgph.0000285.s011.pdf]

S6 Table. Association between the IRS and microscopic *P. falciparum* infection prevalence at the end of the wet seasons.

	Microscopic *P. falciparum* infection [a]			
	Unadjusted		Adjusted [b]	
Factor	OR (95% CI)	*p*-value	aOR (95% CI)	*p*-value
**IRS/Survey**				
Pre-IRS (Survey 1, October 2012)	1.00	-	1.00	-
Post-IRS (Survey 3, October 2015)	0.51 (0.45-0.58)	< 0.001	0.47 (0.40-0.54)	< 0.001
**Age groups**				
1-5 years	1.00	-	1.00	-
6-10 years	2.31 (1.88-2.85)	< 0.001	2.27 (1.84-2.79)	< 0.001
11-20 years	1.58 (1.29-1.94)	< 0.001	1.52 (1.23-1.89)	< 0.001
21-39 years	0.61 (0.48-0.79)	< 0.001	0.57 (0.44-0.74)	< 0.001
≥ 40 years	0.66 (0.52-0.82)	< 0.001	0.63 (0.50-0.80)	< 0.001
**Sex**				
Female	1.00	-	1.00	-
Male	1.44 (1.25-1.66)	< 0.001	1.27 (1.10-1.47)	0.001
**Catchment area**				
Vea/Gowrie	1.00	-	1.00	-
Soe	1.22 (1.06-1.41)	0.005	1.24 (1.08-1.44)	0.003
**LLIN usage (previous night)**				
No	1.00	-	1.00	-
Yes	0.76 (0.61-0.95)	0.015	0.79 (0.62-1.01)	0.058
**Antimalarial treatment (previous 2-weeks)**				
No treatment	1.00	-	1.00	-
Treatment	1.11 (0.96-1.28)	0.174	0.86 (0.73-1.01)	0.060

OR=odds ratio; aOR=adjusted odds ratio; CI=confidence interval, to deal with the repeated measures the cluster sandwich variance estimator was used

[a] Participants were excluded from the model if their antimalarial treatment in the previous two weeks was not known: Survey 3 (N = 79).

[b] Age group, sex, catchment area, LLIN usage the previous night, and antimalarial treatment in the previous two weeks are adjusted for in the multivariable logistic regression model.
