# Supplementary material for: Indoor residual spraying with a non-pyrethroid insecticide reduces the reservoir of Plasmodium falciparum in a high-transmission area in northern Ghana
Source: PLOS Glob Public Health. 2022 May 18;2(5):e0000285. doi: 10.1371/journal.pgph.0000285 (PMC9121889; doi:10.1371/journal.pgph.0000285)
Supplement: S7 Table — (PDF) [file pgph.0000285.s012.pdf]

S7 Table. Association between the IRS and microscopic *P. falciparum* infection prevalence at the end of the dry seasons.

	Microscopic *P. falciparum* infection [a]			
	Unadjusted		Adjusted [b]	
**Factor**	OR (95% CI)	*p*-value	aOR (95% CI)	*p*-value
**IRS/Survey**				
Pre-IRS (Survey 2, May/June 2013)	1.00	-	1.00	-
Post-IRS (Survey 4, May/June 2016)	0.41 (0.35-0.47)	< 0.001	0.36 (0.31-0.43)	< 0.001
**Age groups**				
1-5 years	1.00	-	1.00	-
6-10 years	2.34 (1.85-2.96)	< 0.001	2.42 (1.91-3.08)	< 0.001
11-20 years	1.75 (1.37-2.23)	< 0.001	1.86 (1.44-2.39)	< 0.001
21-39 years	0.38 (0.27-0.54)	< 0.001	0.37 (0.26-0.53)	< 0.001
≥ 40 years	0.38 (0.27-0.52)	< 0.001	0.38 (0.28-0.53)	< 0.001
**Sex**				
Female	1.00	-	1.00	-
Male	1.53 (1.29-1.80)	< 0.001	1.32 (1.11-1.58)	0.002
**Catchment area**				
Vea/Gowrie	1.00	-	1.00	-
Soe	1.23 (1.04-1.45)	0.013	1.33 (1.12-1.59)	0.001
**LLIN usage (previous night)**				
No	1.00	-	1.00	-
Yes	0.88 (0.71-1.10)	0.264	1.03 (0.81-1.30)	0.825
**Antimalarial treatment (previous 2 weeks)**				
No treatment	1.00	-	1.00	-
Treatment	0.56 (0.40-0.79)	0.001	0.53 (0.37-0.76)	< 0.001

OR=odds ratio; aOR=adjusted odds ratio; CI=confidence interval, to deal with the repeated measures the cluster sandwich variance estimator was used
[a] Participants were excluded from the model if their antimalarial treatment in the previous two weeks was not known: Survey 4 (N = 12)
[b] Age group, sex, catchment area, LLIN usage the previous night, and antimalarial treatment in the previous two weeks are adjusted for in the multivariable logistic regression model.
