# Supplementary material for: Indoor residual spraying with a non-pyrethroid insecticide reduces the reservoir of Plasmodium falciparum in a high-transmission area in northern Ghana
Source: PLOS Glob Public Health. 2022 May 18;2(5):e0000285. doi: 10.1371/journal.pgph.0000285 (PMC9121889; doi:10.1371/journal.pgph.0000285)
Supplement: S8 Table — The reference for all comparisons was Survey 1 (pre-IRS, October 2012). (PDF) [file pgph.0000285.s013.pdf]

S8 Table. Stratum-specific estimates for the association between the IRS and microscopic *P. falciparum* prevalence at the end of the wet seasons. The reference for all comparisons was Survey 1 (pre-IRS, October 2012).

	Microscopic *P. falciparum* infection [a]		
	**Pre-IRS Survey 1 (October 2012)**	**Post-IRS Survey 3 (October 2015)**	
**Factor**	aOR [b]	aOR (95% CI) [b]	*p*-value
**Age groups**			
1-5 years	1.00	0.19 (0.13-0.27)	< 0.001
6-10 years	1.00	0.41 (0.31-0.56)	< 0.001
11-20 years	1.00	0.56 (0.42-0.73)	< 0.001
21-39 years	1.00	0.63 (0.43-0.94)	0.023
≥ 40 years	1.00	0.83 (0.59-1.18)	0.311

aOR=adjusted odds ratio; CI=confidence interval, to deal with the repeated measures the cluster sandwich variance estimator was used

[a] Participants were excluded from the model if their antimalarial treatment in the previous two weeks was not known: Survey 3 (N = 79).

[b] Age group, sex, catchment area, LLIN usage the previous night, and antimalarial treatment in the previous two weeks are adjusted for in the multivariable logistic regression model.
