# Supplementary material for: Indoor residual spraying with a non-pyrethroid insecticide reduces the reservoir of Plasmodium falciparum in a high-transmission area in northern Ghana
Source: PLOS Glob Public Health. 2022 May 18;2(5):e0000285. doi: 10.1371/journal.pgph.0000285 (PMC9121889; doi:10.1371/journal.pgph.0000285)
Supplement: S9 Table — The reference for all comparisons was Survey 2 (pre-IRS, May/June 2013). (PDF) [file pgph.0000285.s014.pdf]

S9 Table. Stratum-specific estimates for the association between the IRS and microscopic *P. falciparum* infection prevalence at the end of the dry seasons. The reference for all comparisons was Survey 2 (pre-IRS, May/June 2013).

	Microscopic *P. falciparum* infection [a]		
	**Pre-IRS Survey 2 (May/June 2013)**	**Post-IRS Survey 4 (May/June 2016)**	
**Factor**	aOR [b]	aOR (95% CI) [b]	*p*-value
**Age groups**			
1-5 years	1.00	0.21 (0.13-0.33)	< 0.001
6-10 years	1.00	0.50 (0.38-0.68)	< 0.001
11-20 years	1.00	0.34 (0.25-0.46)	< 0.001
21-39 years	1.00	0.25 (0.12-0.52)	< 0.001
≥ 40 years	1.00	0.39 (0.23-0.65)	< 0.001
**Catchment area**			
Vea/Gowrie	1.00	0.24 (0.19-0.31)	< 0.001
Soe	1.00	0.49 (0.39-0.61)	< 0.001

aOR=adjusted odds ratio; CI=confidence interval, to deal with the repeated measures the cluster sandwich variance estimator was used

[a] Participants were excluded from the model if their antimalarial treatment in the previous two weeks was not known: Survey 4 (N = 12).

[b] Age group, sex, catchment area, LLIN usage the previous night, and antimalarial treatment in the previous two weeks are adjusted for in the multivariable logistic regression model.
