# Supplementary material for: Indoor residual spraying with a non-pyrethroid insecticide reduces the reservoir of Plasmodium falciparum in a high-transmission area in northern Ghana
Source: PLOS Glob Public Health. 2022 May 18;2(5):e0000285. doi: 10.1371/journal.pgph.0000285 (PMC9121889; doi:10.1371/journal.pgph.0000285)
Supplement: S10 Table — (PDF) [file pgph.0000285.s015.pdf]

S10 Table. Association between the IRS and *P. falciparum* infection (i.e., microscopic or submicroscopic) prevalence at the end of the wet seasons.

	P. falciparum infection (i.e., microscopic or submicroscopic) [a]			
	Unadjusted		Adjusted [b]	
Factor	OR (95% CI)	*p*-value	aOR (95% CI)	*p*-value
**IRS/Survey**				
Pre-IRS (Survey 1, October 2012)	1.00	-	1.00	-
Post-IRS (Survey 3, October 2015)	0.25 (0.22-0.29)	< 0.001	0.22 (0.19-0.26)	< 0.001
**Age groups**				
1-5 years	1.00	-	1.00	-
6-10 years	2.49 (2.01-3.09)	< 0.001	2.69 (2.15-3.35)	< 0.001
11-20 years	2.49 (2.02-3.06)	< 0.001	2.79 (2.22-3.50)	< 0.001
21-39 years	1.14 (0.92-1.42)	0.245	1.08 (0.85-1.37)	0.546
≥ 40 years	1.07 (0.87-1.30)	0.529	1.05 (0.84-1.31)	0.692
**Sex**				
Female	1.00	-	1.00	-
Male	1.43 (1.25-1.63)	< 0.001	1.37 (1.18-1.59)	< 0.001
**Catchment area**				
Vea/Gowrie	1.00	-	1.00	-
Soe	1.60 (1.40-1.83)	< 0.001	1.71 (1.47-1.98)	< 0.001
**LLIN usage (previous night)**				
No	1.00	-	1.00	-
Yes	0.72 (0.58-0.90)	0.004	0.82 (0.64-1.05)	0.110
**Antimalarial treatment (previous 2 weeks)**				
No treatment	1.00	-	1.00	-
Treatment	1.35 (1.17-1.56)	< 0.001	0.88 (0.74-1.04)	0.141

OR=odds ratio; aOR=adjusted odds ratio; CI=confidence interval, to deal with the repeated measures the cluster sandwich variance estimator was used

[a] Participants were excluded from the model if their (i) antimalarial treatment in the previous two weeks was not known: Survey 3 (N = 79) and/or (ii) the participant dried blood spot was not available for PCR (N=4). Age group, sex, catchment area, LLIN usage the previous night, antimalarial treatment in the previous two weeks.

[b] Age group, sex, catchment area, LLIN usage the previous night, and antimalarial treatment in the previous two weeks are adjusted for in the multivariable logistic regression model.
