# Supplementary material for: Indoor residual spraying with a non-pyrethroid insecticide reduces the reservoir of Plasmodium falciparum in a high-transmission area in northern Ghana
Source: PLOS Glob Public Health. 2022 May 18;2(5):e0000285. doi: 10.1371/journal.pgph.0000285 (PMC9121889; doi:10.1371/journal.pgph.0000285)
Supplement: S11 Table — Results are expressed in terms of Attributable Risk (AR) and Attributable Risk percentage (AR%). (PDF) [file pgph.0000285.s016.pdf]

S11 Table. Absolute decrease in the probability of having a *P. falciparum* infection (i.e., microscopic or submicroscopic) post-IRS in Bongo at the end of the wet season. Results are expressed in terms of Attributable Risk (AR) and Attributable Risk percentage (AR%).

Outcome	Demographic characteristics	Pre-IRS to Post-IRS End of wet season Survey 1 to Survey 3 AR (95% CI)	AR% (95% CI)	*p*-value
Positive for a *P. falciparum* infection (i.e., microscopic or submicroscopic)	**All**	0.322 (0.293, 0.351)	43.6 (40.3, 46.8)	< 0.001
	**Age Groups**			
	1-5 years	0.466 (0.403, 0.529)	65.1 (58.1, 70.9)	< 0.001
	6-10 years	0.298 (0.238, 0.358)	35.3 (28.7, 41.4)	< 0.001
	11-20 years	0.277 (0.220, 0.334)	32.9 (26.6, 38.7)	< 0.001
	21-39 years	0.302 (0.227, 0.377)	46.5 (36.3, 55.1)	< 0.001
	≥ 40 years	0.290 (0.227, 0.354)	46.1 (37.4, 53.5)	< 0.001
	**Sex**			
	Female	0.315 (0.274, 0.355)	45.1 (40.2, 49.6)	< 0.001
	Male	0.330 (0.288, 0.372)	42.0 (37.3, 46.4)	< 0.001
	**Catchment area**			
	Vea/Gowrie	0.288 (0.245)	43.1 (37.7, 48.1)	< 0.001
	Soe	0.351 (0.312, 0.391)	43.8 (39.4, 31.3)	< 0.001

AR (Attributable Risk = (Pre-IRS Risk) - (Post-IRS Risk))
AR% (Attributable Risk Percentage = ((Pre-IRS Risk) - (Post-IRS Risk)/ (Pre-IRS Risk)) * 100%)
CI=confidence interval
