# Supplementary material for: Indoor residual spraying with a non-pyrethroid insecticide reduces the reservoir of Plasmodium falciparum in a high-transmission area in northern Ghana
Source: PLOS Glob Public Health. 2022 May 18;2(5):e0000285. doi: 10.1371/journal.pgph.0000285 (PMC9121889; doi:10.1371/journal.pgph.0000285)
Supplement: S12 Table — The reference for all comparisons was Survey 1 (pre-IRS, October 2012). (PDF) [file pgph.0000285.s017.pdf]

S12 Table. Stratum-specific estimates for the association between the IRS and *P. falciparum* (i.e., microscopic or submicroscopic) infection prevalence at the end of the wet seasons. The reference for all comparisons was Survey 1 (pre-IRS, October 2012).

	P. falciparum infection (i.e., microscopic or submicroscopic) [a]		
	**Pre-IRS Survey 1 (October 2012)**	**Post-IRS Survey 3 (October 2015)**	
**Factor**	aOR [b]	aOR (95% CI) [b]	*p*-value
**Age groups**			
1-5 years	1.00	0.14 (0.10-0.19)	< 0.001
6-10 years	1.00	0.19 (0.13-0.27)	< 0.001
11-20 years	1.00	0.22 (0.16-0.31)	< 0.001
21-39 years	1.00	0.28 (0.20-0.39)	< 0.001
≥ 40 years	1.00	0.30 (0.22-0.41)	< 0.001
**Catchment area**			
Vea/Gowrie	1.00	0.28 (0.23-0.34)	< 0.001
Soe	1.00	0.16 (0.13-0.20)	< 0.001

aOR=adjusted odds ratio; CI=confidence interval, to deal with the repeated measures the cluster sandwich variance estimator was used

[a] Participants were excluded from the model if their (i) antimalarial treatment in the previous two weeks was not known: Survey 3 (N = 79) and/or (ii) the participant dried blood spot was not available for PCR (N=4).

[b] Age group, sex, catchment area, LLIN usage the previous night, and antimalarial treatment in the previous two weeks are adjusted for in the multivariable logistic regression model.
