# Supplementary material for: Indoor residual spraying with a non-pyrethroid insecticide reduces the reservoir of Plasmodium falciparum in a high-transmission area in northern Ghana
Source: PLOS Glob Public Health. 2022 May 18;2(5):e0000285. doi: 10.1371/journal.pgph.0000285 (PMC9121889; doi:10.1371/journal.pgph.0000285)
Supplement: S13 Table — (PDF) [file pgph.0000285.s018.pdf]

S13 Table. The estimated number of *P. falciparum* genomes.

	Estimated number of *P. falciparum* genomes [a]			
	Pre-IRS		Post-IRS	
	Survey 1<br>End of wet season<br>(October 2012)	Survey 2<br>End of dry season<br>(May/June 2013)	Survey 3<br>End of wet season<br>(October 2015)	Survey 4<br>End of dry season<br>(May/June 2016)
Survey	2,624	1,637	1,083	806
Age groups				
1-5 years	683 (26.0)	423 (25.8)	120 (11.1)	65 (8.1)
6-10 years	961 (36.6)	634 (38.7)	401 (37.0)	408 (50.6)
11-20 years	603 (23.0)	448 (27.4)	355 (32.8)	279 (34.6)
21-39 years	175 (6.7)	56 (3.4)	76 (7.0)	21 (2.6)
≥ 40 years	202 (7.7)	76 (4.6)	131 (12.1)	33 (4.1)

[a] MOI_{var} used to estimate the number of diverse *P. falciparum* genomes per isolates (see Methods for additional details). Data reflect the number (% (n/N)) of subjects.
