# Supplementary material for: Indoor residual spraying with a non-pyrethroid insecticide reduces the reservoir of Plasmodium falciparum in a high-transmission area in northern Ghana
Source: PLOS Glob Public Health. 2022 May 18;2(5):e0000285. doi: 10.1371/journal.pgph.0000285 (PMC9121889; doi:10.1371/journal.pgph.0000285)
Supplement: S1 Text — (PDF) [file pgph.0000285.s019.pdf]

S1 Text. Structured questionnaire

Indoor residual spraying with a non-pyrethroid insecticide reduces the reservoir of *Plasmodium falciparum* in a high-transmission area in northern Ghana

Kathryn E. Tiedje[¶], Abraham R. Oduro[¶], Oscar Bangre, Lucas Amenga-Etego, Samuel K Dadzie, Maxwell A Appawu, Kwadwo Frempong, Victor Asoala, Shazia Ruybal-Pésantez, Charles A. Narh, Samantha L. Deed, Dionne C. Argyropoulos, Anita Ghansah, Samuel A. Agyei, Sylvester Segbaya, Kwame Desewu, Ignatius Williams, Julie A. Simpson, Keziah Malm, Mercedes Pascual, Kwadwo A. Koram, and Karen P. Day*

[¶] KET and ARO contributed equally to this work as first authors.

* Corresponding author: Karen P. Day; karen.day@unimelb.edu.au

S1 Text. Structured questionnaire.

SECTION 1: PARTICIPANT DATA FORM

1. Study ID	☐☐☐☐☐☐☐	**STUDY_ID**
2. Date surveyed (DD-MM-YYYY)	☐☐☐☐☐☐☐☐	**DATE**
3. Relationship of respondent to study participant	1. Self / 2. Mother / 3. Father / 4. Guardian / 5. Other (specify)	**RELATION**
4. Sex	1. Male / 2. Female	**SEX**
5. Date of birth (**DD-MM-YYYY**)	☐☐☐☐☐☐☐☐	**DOB**
6. Compound Name	________________________________	**CPD_NAM**
7. Compound ID	☐☐☐☐☐☐	**CPD_ID**
8. Name of village	________________________________	**VILLAGE**
9. Name of Section	________________________________	**SECTION**
10. Educational level of participant	1. None / 2. Prim / 3. JSS/Middle / 4. SSS/Voc / 5. Above SSS	**EDUC**
11. Occupation of participant	1. House wife / 2. Farmer / 3. Trader / 4.Student/pupil / 5.Public/Civil servant / 6. Unemployed / 8. NA / 7. Other (specify)	**OCCUP**

SECTION 2: MALARIA PREVENTION

12. What are you (your HH) doing to prevent you (your ward/child) from getting malaria? ***(circle all mentioned, multiple responses allowed)***

1. Bed nets	2. Repellents	3. Strong scented flowers	**MAL_P**
4. Clean environment	5.Insecticide spray	6. Nothing	
7. Other (specify)			

13. At any time in the past 12 months, has anyone come into your home to spray the interior walls against mosquitoes?

1. Yes	2. No	9. DK	**SPRAY**

14. If YES, who sprayed your home?

1.Government Program	2. Private Company	3. NGO	8. NA	9.DK	
5. Other (specify)					**SPRAYHOME**

15. Does your household have any bed nets that can be used while sleeping?

1. Yes	2. No	**OWNNET**

16. If YES, how many mosquito nets does your household have?

Number:	**8. NA**	**NUMNET**

17. Has the bed net been treated with insecticide?

1. Yes	2. No	8. NA	9. DK	**TREATNET**

18. When was the bed net acquired?

1. <6months	2. > 6months	3. >2 years	8. NA	9. DK	**TBNAQUIRE**

19. How was the bed net acquired?

1. Free distribution	2. Purchased	3. ANC	8. NA	9. DK	**HBNAQUIRE**

20. Did you (your ward/child) sleep under a bed net last night?

21. SECTION 1/SECTION 2 completed by

1. Yes	2. No	**SLEEPNET**
		**CODES1S2**

SECTION 3: SYMPTOMATIC SCREENING

22. The stud. Do you (your ward/child) have health insurance?		1. Yes	2. No	**INSURANCE**
23. Is the health insurance still valid /working?	1. Yes	2. No	8. NA	**INSUR_V**
24. Have you used the health insurance this year?	1. Yes	2. No	8. NA	**INSUR_USE**

25. Have you (your child/ward) been sick within the past two weeks?	1. Yes	2. No	**SICK**

IF THE RESPONSE TO QUESTION 25 IS <u>NO</u>, SKIP DIRECTLY TO SECTION 4

26. If you (your ward/child) were sick within the **PAST TWO WEEKS,** did you have any of the following symptoms?

a. Fever	1. Yes	2. No	8. NA	**FEVER**
b. Headache	1. Yes	2. No	8. NA	**HEADACHE**
c. Chills	1. Yes	2. No	8. NA	**CHILL**
d. Rigors	1. Yes	2. No	8. NA	**RIGORS**
e. Convulsion	1. Yes	2. No	8. NA	**CONVULSE**
f. Cough	1. Yes	2. No	8. NA	**COUGH**
g. Ear pain/discharge	1. Yes	2. No	8. NA	**EARPAIN**
h. Diarrhea	1. Yes	2. No	8. NA	**DIARRHOEA**
i. Other (Specify)				**OTHER**

27. Did you (your child/ward) seek treatment for the illness?	1. Yes	2. No	8. NA	**SEEKT**

IF THE RESPONSE TO QUESTION270 IS <u>NO</u>, SKIP DIRECTLY TO SECTION 4

28. Where did you (your child/ward) go for treatment during this illness? ***(circle all mentioned, multiple responses allowed)***

1. Home	2. Hospital	3. Health Centre/ Clinic	4. CHPs compound	**FACILITY**
5. Traditional healer/ Herbalist		6. Drug store /chemical seller	8. NA	

29. Was blood taken from your (your ward/child's) finger/heel for testing?	1. Yes	2. No	**BLDTEST**

30. Were you (your ward/child) given a malaria treatment?	1. Yes	2. No	9. DK	**MAL_T**

31. If YES to malaria treatment, what antimalarial treatment were you (your child/ward) given?

1. ASAQ	2. AL	3. DHP	4. SP	5. CQ	**ANTIMAL**
6.Quinine	7. AS	10. AQ	11. Other		
9. DK	8.NA				

32. SECTION 3 completed by [ ][ ] **CODES3**

SECTION 4: PHYSICAL EXAMINATION

33. Auxiliary Temperature (^{o} C): [ ][ ] • [ ] **TEMP**

34. Weight (kg): [ ][ ] • [ ] **WEIGHT**

35. Systolic blood pressure (mmHg): [ ][ ][ ] **SBP**

36. Diastolic blood pressure (mmHg): [ ][ ][ ] **DBP**

37. SECTION 4 completed by [ ][ ] **CODES4**

SECTION 5: SPECIMENS COLLECTED AT ENROLMENT

	SPECIMEN			
38.	Filter paper blood blot	1. Yes	2. No	**BBLOT**
39.	Blood smear	1. Yes	2. No	**BSMEAR**

LABORATORY RESULTS

40. Haemoglobin concentration (g/dl) [ ][ ] • [ ] **Hgb**

41. Rapid Malaria Test (RDT)

1. Positive	2. Negative	3. Not done	**RAMAT**

42. SECTION 5 completed by [ ][ ] **CODES5**
