# Supplementary material for: Indoor residual spraying with a non-pyrethroid insecticide reduces the reservoir of Plasmodium falciparum in a high-transmission area in northern Ghana
Source: PLOS Glob Public Health. 2022 May 18;2(5):e0000285. doi: 10.1371/journal.pgph.0000285 (PMC9121889; doi:10.1371/journal.pgph.0000285)
Supplement: Attachment — Submitted filename: PGPH-D-21-00825R1 Response to Reviewers 110322.pdf [file pgph.0000285.s020.pdf]

RESPONSES TO REVIEWERS FOR PGPH-D-21-00825:
"Indoor residual spraying with a non-pyrethroid insecticide reduces the reservoir of *Plasmodium falciparum* in a high-transmission setting in northern Ghana"

REVIEWER #1

A nicely done paper looking at combining ento and epi indicators to look at impact of vector control. The findings are interesting and informative for program managers deciding about vector control and also for programs that might be thinking about moving towards elimination. I was particularly interested in the findings regarding residual reservoir of p. falciparum among the older population as this group gets less attention through traditional control measures. The fact that they still have this reservoir despite these vector control interventions highlights the need for more radical approaches in the move to elimination. Well done.

General comments:

1. **When showing differences or changes in values, frequently no p. value was included in the narrative (though it was shown in the graphics). I believe best practice is to include the p value in the narrative report of the data points as well so that the reader can see immediately whether the difference was significant. This happened in several areas throughout the results section.**

We thank the reviewer for this comment and wish to provide clarifications regarding our use of p-values in the Results. For the comparisons presented in the results (e.g., prevalence of P. falciparum infections, S4 Table and Figure 3), no p-values were reported since these metrics were provided to describe overall trends/patterns observed in the data pre- to post-IRS that were subsequently investigated after adjusting for potential confounders identified *a priori* (see Methods). When reporting these findings p-values were included as these were the primary analyses investigating the association between the IRS and *P. falciparum* infection (i.e., microscopic and/or sub microscopic). All p-values for these analyses are provided in Tables (manuscript and supplement) and referenced appropriately in the manuscript.

We have checked that none of our graphics (i.e., Figs. in the manuscript or supplement) have p-values included in the image. However, we did include with the Fig 3 and Fig 4 titles the phrase "Significant reductions in the prevalence …". We understand that this is inconsistent and doesn't match with the narrative in the results. We have now edited Fig 3 (Line 436) and Fig 4 (Line 516) titles as follows "Reductions in the prevalence….", removing the word "significant" so that we are consistent with our use of p-values as discussed above and laid out in the results.

2. **Did you collect any data on cost? There needs to be some recognition of the extra cost of IRS and nets in the same venues. I realize this study occurred some years ago, but the more recent move to more expensive insecticides for both IRS and ITN make at least addressing the cost issue important.**

The cost of combining vector control strategies was outside the scope of this research but is something the NMCP/GHS is investigating. Previous research from our colleagues in Ghana has shown that LLINs and IRS are impactful and cost-effective malaria-control interventions for reducing morbidity and/or mortality in children (1–4). They however have not investigated the costs and cost-effectiveness of these interventions on the reservoir of infection. To address this important comment raised by the reviewer, we have added specific text to the Discussion (Line 627-631: "Although not undertaken as part of this study, additional research by NMCPs on the costs and cost-effectiveness would be valuable to estimate the financial implications of combining IRS and LLINs long-term to not only reduce morbidity and mortality but also to decrease the size of the reservoir of infection. Such analyses are fiscally prudent with the shift towards use of more expensive insecticides.*")*

Specific comments:

Line 95-98: the assertion that entomological surveillance isn't done is not true. This is routinely part of malaria control program and donors actually require some basic amount of reporting. Please revise to more accurately reflect the situation.

This paragraph in the Introduction has been revised to take into account the recommendations of the reviewer (Line 94-97: "During programmatic scale up, entomological and parasitological surveillance of these interventions under operational conditions rarely occurs, and never simultaneously, as once they are found to be effective, funding to support ongoing monitoring and evaluation is time-limited.").

Line 173: Were no new nets distributed between 2010-12 and 2016? Net durability is estimated at <3 years and there may have been deterioration. This at least needs to be discussed/addressed in the paper.

We thank the reviewer for this question. In both 2010-2012 and 2016, universal coverage campaigns (UCC) were undertaken to distribute LLINs across the Upper East Region of Ghana, including Bongo District. Between 2012 and 2016, LLINs were still freely disturbed in Bongo by the Ministry of Health using routine services like antenatal care (ANC) and Expanded Programme on Immunization (EPI) visits, as well as school distributions (5). This routine distribution of LLINs in Bongo District between 2012-2016 was undertaken so that net coverage remained high and so that damaged and/or older LLINs where the active insecticides may no longer be bioactive were replaced. We have clarified these important details as suggested by the reviewer in the Methods "Vector control interventions" section (Line 179-187).

Line 194: How did you arrive at 2000 for the survey sample? There is reference to another paper, but some of these details should also be included in this one for readers who only look at one paper.

As requested, we have now included the previously published study design details with this manuscript as suggested for clarity in the Methods "Study design and procedures" section (Line 227-247).

Line 369: Treatment. What is the question used on the questionnaire to determine treatment? In many places providers don't treat for malaria as often in the dry season as they assume the fever is something else and don't test (which is the protocol, but sometimes doesn't happen). Does your questionnaire look at 'treatment seeking' or actual treatment received? It would be good to clarify here. Also, did you look at any of the surveillance data to look at trends in the district and whether they match your survey data?

We thank the reviewer for these questions and have now provided a copy of the questionnaire (see S1 Appendix) used for this study. In this study we specifically asked all participants if they had (1) been sick in the previous 2-weeks. If they responded "yes", we then asked if they (2) sought treatment for the illness. If they responded "yes" to seeking treatment we asked if they (3) were given an antimalarial treatment. The data presented in S1 Table (i.e., "Antimalarial treatment (previous 2-weeks)" represent those participants that were (1) sick, (2) sought treatment, and (3) were provided with an antimalarial treatment in the previous 2-weeks. We have now clarified this as recommended in the Methods "Study design and procedures" section (Line 221-225) as well as in the footnotes included for S4 Table.

It is our intention with our NMCP/GHS colleagues to address this information on morbidity (and mortality) in a manuscript currently under preparation reporting on 6-years of data covering both the IRS and SMC interventions in Bongo District.

REVIEWER #2

The paper describes a well-designed observational study that looked at the impact of IRS in addition to ITNs on malaria infection prevalence, molecular complexity of infection, infection densities, and some elements of vectorial capacity. The interrupted time series approach is appropriate, and the analytical approach makes sense. In addition, the results and conclusions are compelling.

Line 172: Which brand and type of LLINs were distributed in the 2016 campaign? Any measures of how many were distributed and what levels of coverage were achieved?

In 2016 through a universal coverage campaign (UCC), PermaNet 2.0 (deltamethrin, Vestergaard-Frandsen), Olyset (permethrin, Sumitomo Chemical Co.), or DawaPlus 2.0 (deltamethrin, TANA Netting) were distributed in Bongo, which are the most widely distributed nets in Ghana. Following this 2016 campaign LLIN coverage (i.e., household with at least one net) for the Upper East Region was reported to be 93.9% (5). We have included this information in the Methods "Vector control interventions" section (Line 176-187).

Line 190: How was IRS coverage determined? Does this represent the standard "eligible structures sprayed/eligible structures encountered," from operational reports?

The IRS coverage data recorded in the manuscript currently is that collected from the participants surveyed using the structured questionnaires (see Line 213-215). Given the reviewer's question we have clarified this reported IRS coverage and have included additional information in the Methods "Vector control interventions" based on AGAMal's operational reports (see Line 203-210).

Line 194: Some discussion of why there were no non-IRS (control) communities included in the interrupted time series, with an acknowledgement that it could have made for a stronger study design, would be helpful.

This interrupted time-series study in Bongo was observational and undertaken to investigate the impact of adding IRS to LLINs on the asymptomatic *P. falciparum* reservoir, in a programmatic context. As pointed out by Katureenbe et al. (2016), once these interventions are included as part of a country's NMCP, little high-quality contemporary evidence is collected in operational settings (6). Hence one of the primary advantages of this comprehensive study in Bongo, is that we were able to monitor the impacts of adding IRS to LLINs under operational conditions at the population-level, thus helping to fill this gap in the current literature.

Line 214: I think specimens were identified to the species group (sensu lato) level, no?

The main vectors were identified to the *Anopheles* species level as *An. gambiae* s.l. and *An. funestus.* We have now clarified this in the Methods "Entomological parameters" section (Line 255-256).

Line 216: Are the EIRs presented as An. gambiae s.l.-specific rates, or are these estimates aggregated to all Anopheles spp?

The EIR were presented as aggregated rates for both *An. gambiae* s.l., which is the main vector, and *An. funestus*, the minor vector. We have now clarified this in the Methods "Entomological parameters" section (Line 258-263) and Fig. 2.

Line 238: Why were symptomatic participants excluded from the analysis?

Participants who were symptomatic for a *P. falciparum* infection were relatively rare and excluded from the analyses since they did not meet the inclusion criteria as defined in our approved ethics applications. We were studying asymptomatic infections.

Line 301: Some specifics about the statistical analysis plan and experimental design are needed. How many surveys were conducted at each time point? What was the expected effect size? Power and/or sample size considerations?

As requested, we have now included the previously published study design details with this manuscript as suggested for clarity in the Methods "Study design and procedures" section (Line 227-247). In addition, the 2,000 participants per survey was sufficient to detect a risk ratio ≤ 0.85 for *P. falciparum* prevalence between the pre-IRS (i.e., unexposed) and post-IRS (i.e., exposed) with a 95% confidence level, 80% power, and a 1:1 sample ratio between the pre- and post-IRS surveys (EWS and EDS).

Line 427: It's not clear what the expected effect on parasite density is? How would decreased transmission lead to lower (or higher) density infections?

The simple explanation comes from our observation that IRS reduced MOI and this in turn led to lower parasite densities. The observed reduction in MOI is a consequence of a decrease in new infections and clearance of existing infections (addressed in the Discussion Line 609-610), which in turn could lead to the observed reductions in *P. falciparum* density pre-to post-IRS at the end of the wet season (see Fig 2 and S4 Table).

REVIEWER RESPONSE REFERENCES:

1. Smith Paintain L, Awini E, Addei S, Kukula V, Nikoi C, Sarpong D, et al. Evaluation of a universal long-lasting insecticidal net (LLIN) distribution campaign in Ghana: Cost effectiveness of distribution and hang-up activities. Malar J. 2014;13(1):1–13.
2. Johns B, Haile M. PMI IRS country programs: 2020 comparative cost analysis [Internet]. Rockville, MD; Available from: https://www.pmi.gov/docs/default-source/default-document-library/implementing-partner-reports/africa-indoor-residual-spraying-project-pmi-irs-country-programs-2014-comparative-cost-analysis.pdf?sfvrsn=4
3. IVCC. Evidence snapshot: cost and cost-effectiveness of 3rd generation IRS (3GIRS). 2019.
4. Abuaku B, Ahorlu C, Psychas P, Ricks P, Oppong S, Mensah S, et al. Impact of indoor residual spraying on malaria parasitaemia in the Bunkpurugu-Yunyoo District in northern Ghana. Parasit Vectors. 2018;11(1):1–11.
5. Gogue C, Wagman J, Tynuv K, Saibu A, Yihdego Y, Malm K, et al. An observational analysis of the impact of indoor residual spraying in Northern, Upper East, and Upper West Regions of Ghana: 2014 through 2017. Malar J [Internet]. 2020;19(1):1–13. Available from: https://doi.org/10.1186/s12936-020-03318-1
6. Katureebe A, Zinszer K, Arinaitwe E, Rek J, Kakande E, Charland K, et al. Measures of Malaria Burden after Long-Lasting Insecticidal Net Distribution and Indoor Residual Spraying at Three Sites in Uganda: A Prospective Observational Study. PLoS Med. 2016;13(11):1–22.

NOTE: Three additional references have been added to the manuscript to address the questions/comments raised by the reviewers.

1. WHO Global Malaria Programme. Achieving and maintaining universal coverage with long-lasting insecticidal nets for malaria control [Internet]. WHO. 2017. Available from: http://www.who.int/malaria/publications/atoz/who_recommendation_coverage_llin/en/
2. Bhatt S, Weiss DJ, Mappin B, Dalrymple U, Cameron E, Bisanzio D, et al. Coverage and system efficiencies of insecticide-treated nets in Africa from 2000 to 2017. Elife. 2015;4:1–37
3. Ruybal-Pesántez S, Tiedje KE, Pilosof S, Tonkin-Hill G, He Q, Rask TS, et al. Age-specific patterns of DBLa var diversity can explain why residents of high malaria transmission areas remain susceptible to Plasmodium falciparum blood stage infection throughout life. Int J Parasitol. 2022;(in press).
